# The metallic compound promotes primordial follicle activation and ameliorates fertility deficits in aged mice

**DOI:** 10.7150/thno.82553

**Published:** 2023-05-21

**Authors:** Lincheng Han, Yingying Huang, Biao Li, Weiyong Wang, Yan-li Sun, Xiaodan Zhang, Wenbo Zhang, Shuang Liu, Wenjun Zhou, Wei Xia, Meijia Zhang

**Affiliations:** 1 Division of Cell, Developmental and Integrative Biology, School of Medicine, South China University of Technology, Guangzhou, Guangdong 510006, China.; 2 Department of Reproductive Medicine Centre, Guangzhou First People's Hospital, South China University of Technology, Guangzhou, Guangdong 510180, China.

**Keywords:** metallic compounds, primordial follicle activation, fertility, mTOR, Akt

## Abstract

**Background:** Aged women and premature ovarian insufficiency (POI) patients have residual dormant primordial follicles that are hard to be activated through a physiological process. However, there are no effective and safe drugs to help them.

**Methods:** We used the *in vitro* culture model of newborn mouse ovaries to identify the drugs that promote primordial follicle activation and study its mechanisms. It was verified by *in vivo* injection model of newborn mice and *in vitro* culture model of human ovarian tissue. In addition, we used the aged mice as a low infertility model to verify the effects of primordial follicle activation, and fertility by drugs.

**Results:** Eleven metallic compounds activated mouse primordial follicles, and the five most effective compounds were selected for further study. Thapsigargin (TG), CrCl_3_, MnCl_2_, FeCl_3_ and ZnSO_4_ increased the levels of the glycolysis-related proteins (glucose transporter type 4, GLUT4; hexokinase 1, HK1; pyruvate kinase M2, PKM2; phosphofructokinase, liver type, PFKL), phosphorylated mammalian target of rapamycin (p-mTOR) in cultured mouse ovaries. The compound-promoted p-mTOR levels could be completely blocked by 2-DG (the inhibitor of glycolysis). The compounds also increased the levels of phosphorylated protein kinase B (p-Akt). TG-, CrCl_3_- and FeCl_3_-promoted p-Akt levels, but not MnCl_2_- and ZnSO_4_- promoted p-Akt levels, could be completely blocked by ISCK03 (the inhibitor of proto-oncogenic receptor tyrosine kinase, KIT). The injection of newborn mice with the compounds also activated primordial follicles and increased the levels of the glycolysis-related proteins, p-mTOR, and p-Akt. The oral administration of the compounds in adolescent and aged mice promoted primordial follicle activation, and had no obvious side effect. Importantly, ZnSO_4_ also increased ovulated oocytes, oocyte quality and offspring in aged mice. Furthermore, the compounds promoted human primordial follicle activation and increased the levels of the glycolysis-related proteins, p-mTOR, and p-Akt.

**Conclusion:** The metallic compounds activate primordial follicles through the glycolysis-dependent mTOR pathway and/or the PI3K/Akt pathway, and the oral administration of ZnSO_4_ enhances fertility in aged mice. We suggest that these metallic compounds may be oral drugs to ameliorate fertility deficits in aged women and POI patients.

## Introduction

In mammals, the nonrenewable primordial follicle pool is established in the ovary before or around birth 1. The primordial follicles are progressively recruited into the growing follicle pool through a process called primordial follicle activation 2. The others are maintained in a dormant state for the long-term reproductive lifespan of females 3.

The primordial follicle contains a quiescent oocyte surrounded by a single layer of flat pregranulosa cells 1. Primordial follicle activation includes oocyte growth and pregranulosa cell differentiation from a flat to cubic form 3. The activation of the mammalian target of rapamycin (mTOR) pathway in pregranulosa cells promotes proto-oncogenic receptor tyrosine kinase (KIT) ligand (KITL) expression, which binds to KIT to activate the phosphoinositide 3-kinase/protein kinase B (PI3K/Akt) pathway in oocytes 2, 4. Subsequently, forkhead box O3a (FOXO3a) is phosphorylated and translocated from the nucleus to the cytoplasm, resulting in primordial follicle activation 5. Our recent study shows that enhanced glycolysis in pregranulosa cells activates primordial follicles via the mTOR pathway 6. E-cadherin, mitogen-activated protein kinase (MAPK3/1) and histone deacetylase 6 (HDAC6) have been reported to activate primordial follicles by regulating the above signaling pathways 7-9.

The balance between the dormancy and activation of primordial follicles is crucial for the maintenance of female reproductive lifespan 3. In a physiological process, female fertility declines with age beginning in the early 30 s and decreases rapidly beginning at the age of 35, accompanied by the rapid consumption of the primordial follicle pool 10, 11. Notably, the excessive consumption of the primordial follicle pool will lead to premature ovarian insufficiency (POI) 12, 13. The residual dormant primordial follicles in aged women and POI patients are hard to be activated through a physiological process, leading to infertility 13. Traditional assisted reproductive technology (ART) is difficult to apply in aged women and POI patients 11, 13. *In vitro* activation (IVA) by PI3K/Akt stimulators has now been applied clinically, but only several young patients have reproduced their own genetic babies 14. Moreover, the application of IVA has been limited by invasive surgery, low success rate and potential carcinogenicity of stimulators 14. Therefore, it is necessary to explore safer and more effective methods for rescuing the infertility of aged women and POI patients.

Trace elements are crucial for human growth, development and physiology, although they are required in very small quantities 15. In most cases, they enter organisms through diet and act as second messengers to transmit signals in different pathways or form complexes with proteins to perform physiological functions 15. Trace elements are also vital for reproduction. Calcium, zinc and selenium are vital for follicle development, and the lack of iron, zinc, selenium and iodine is associated with infertility 16-19. Importantly, supplementation with calcium, magnesium, chromium, zinc and selenium improves symptoms associated with polycystic ovary syndrome (PCOS) 20, 21. It has also been shown that calcium, chromium, manganese, iron, cobalt, nickel, zinc, arsenic and cadmium can enhance glycolysis and/or glycolysis-related gene expression, and activate the mTOR and PI3K/Akt pathways in various tissues and cells 22-32. In our previous study, lithium activates primordial follicles through the PI3K/Akt pathway 33. Thus, we explored the effects of small molecular compounds containing these elements on the activation of primordial follicles.

In this study, we found that thapsigargin (TG), CrCl_3_, MnCl_2_, FeCl_3_ and ZnSO_4_ activate mouse and human primordial follicles through the glycolysis-dependent mTOR pathway and/or the PI3K/Akt pathway. The oral administration of ZnSO_4_ increases the quantity and quality of ovulated oocytes, and rescues infertility in aged female mice. Thus, we propose that these metallic compounds may be used as oral drugs to promote residual dormant primordial follicle activation in aged women and POI patients.

## Results

### The metallic compounds promote mouse primordial follicle activation *in vitro*

First, we selected seventeen small molecular compounds containing different elements to explore their effects on primordial follicle activation. TG was used to elevate calcium levels by inhibiting Ca^2+^-ATPase activity since extracellular Ca^2+^ has difficulty entering cells 34. We cultured 3 dpp mouse ovaries in the medium supplemented with different drugs for 4 days. Eleven metallic compounds, including AlCl_3_, TG, CrCl_3_, MnCl_2_, FeCl_3_, ZnSO_4_, NaAsO_2_, Na_2_SeO_3_, Na_2_MoO_4_, GdCl_3_ and Pb(CH_3_CO_2_)_2_, increased the number of growing follicles in a dose-dependent manner, and the most effective concentrations were 5 μM, 0.05 μM, 5 μM, 200 μM, 15 μM, 35 μM, 1 μM, 2 μM, 25 μM, 20 μM and 10 μM, respectively (Figure S1-S2). The others, including C_8_H_4_K_2_O_12_Sb_2_ (0-25 μM), CoCl_2_ (0-150 μM), NiCl_2_ (0-150 μM), CuCl_2_ (0-50 μM), CdCl_2_ (0-1 μM) and KIO_3_ (0-150 μM), had no obvious effect on the number of growing follicles (Figure S1-S2).

**Figure 1:**
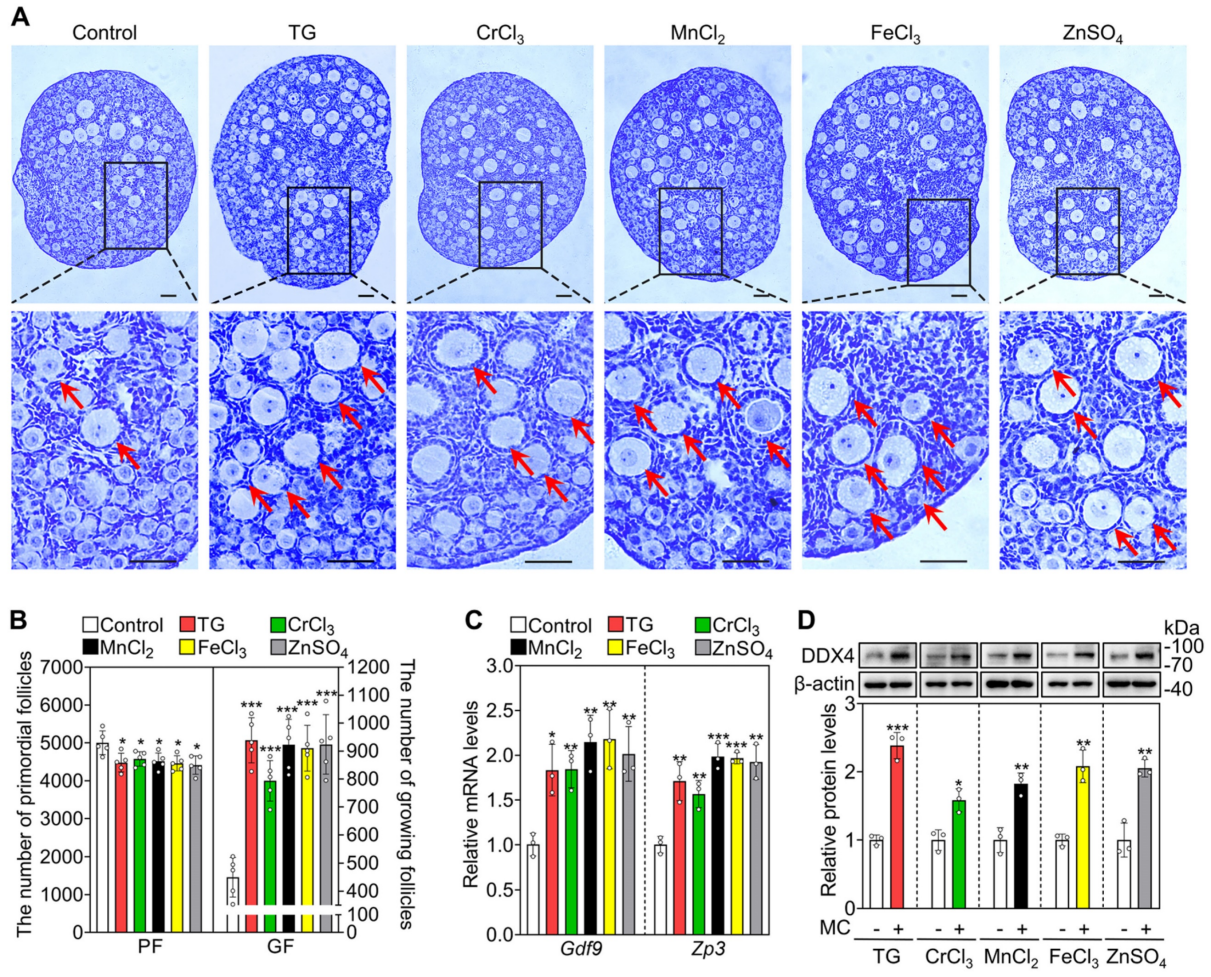
** The metallic compounds promote the activation of primordial follicles in cultured mouse ovaries.** The ovaries from 3 dpp mice were cultured in the medium (control) or the medium supplemented with 0.05 μM TG, 5 μM CrCl_3_, 200 μM MnCl_2_, 15 μM FeCl_3_ or 35 μM ZnSO_4_ for 2 days (**C-D**) or 4 days (**A-B**). **A-B**, Morphological comparison of ovaries (**A**) and the number of primordial follicles (PF) and growing follicles (GF. **B**) in the different treatments. Nuclei was stained with hematoxylin. Red arrows, growing follicles. **C-D**, *Gdf9* and *Zp3* mRNA (**C**) and DDX4 protein levels (**D**) in the different treatments. MC, metallic compounds. All the experiments were independently repeated three or five times, and the representative images are presented. Scale bars, 50 µm. Bars indicate the mean ± SD. *p < 0.05, **p < 0.01, and ***p < 0.001.

**Figure 2:**
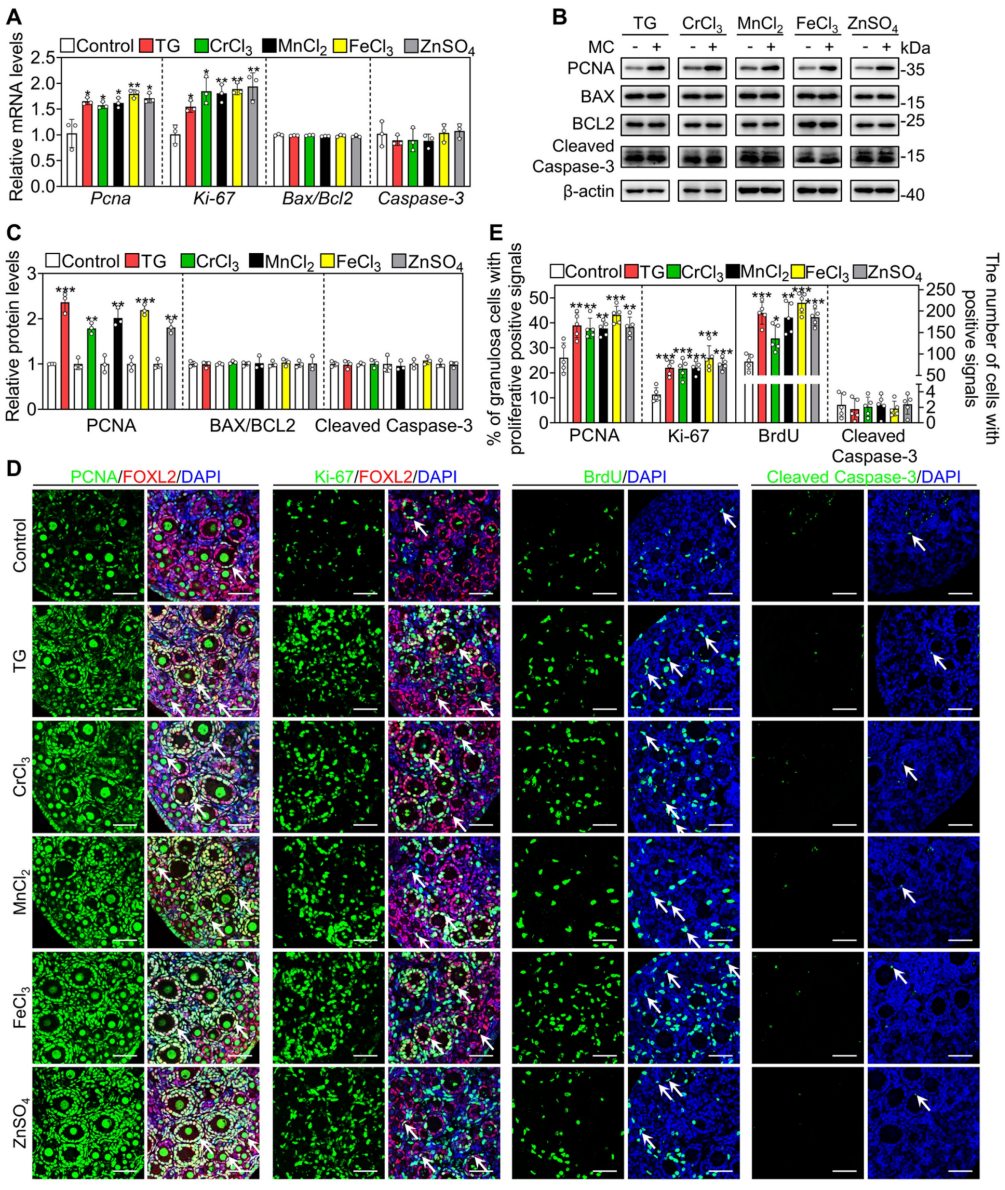
** The metallic compounds promote the proliferation of granulosa cells in cultured mouse ovaries.** The ovaries from 3 dpp mice were cultured in the medium (control) or the medium supplemented with TG, CrCl_3_, MnCl_2_, FeCl_3_ or ZnSO_4_ for 2 days. **A-D**, The mRNA levels of *Pcna*, *Ki-67*, *Bax*/*Bcl-2* and *Caspase-3* (**A**), the protein levels of PCNA, BAX, BCL-2 and Cleaved Caspase-3 (**B-C**), and the immunofluorescence stain of PCNA, Ki-67, BrdU, and Cleaved Caspase-3 (green. **D**) in the different treatments. FOXL2, red; DAPI, blue. **E**, The percentage of granulosa cells with PCNA- and Ki-67-positive signals, and the number of cells with BrdU- and Cleaved Caspase-3-positive signals in the different treatments. All the experiments were independently repeated three or five times, and the representative images are presented. Scale bars, 50 µm. Bars indicate the mean ± SD. *p < 0.05, **p < 0.01, and ***p < 0.001.

Next, we selected the five most effective compounds for further study. Compared with the control group, treatment with TG, CrCl_3_, MnCl_2_, FeCl_3_ and ZnSO_4_ significantly increased the number of growing follicles, the mRNA levels of growth differentiation factor 9 (*Gdf9*) and zona pellucida glycoprotein 3 (*Zp3*, oocyte developmental markers) and the protein levels of DEAD-box helicase 4 (DDX4, a cytosolic marker of oocytes. Figure 1A-D). We further tested granulosa cell proliferation and cell apoptosis. Compared with the control, each of these five compounds significantly increased the mRNA and protein levels of proliferating cell nuclear antigen (PCNA) and/or Ki-67, the percentage of granulosa cells with PCNA- and Ki-67-positive signals, and the number of somatic cells with BrdU-positive signals (Figure 2A-E and S3). However, they had no obvious effect on the mRNA levels of B-cell lymphoma 2-associated X (*Bax*)/B-cell lymphoma 2 (*Bcl-2*) or *Caspase-3*, the protein levels of BAX/BCL-2 or Cleaved Caspase-3, or the number of cells with Cleaved Caspase-3-positive signals (Figure 2A-E and S3). These results demonstrate that these metallic compounds promote mouse primordial follicle activation *in vitro*.

In order to study the subsequent development of the metallic compound-activated follicles, we cultured 3 dpp mouse ovaries in the medium for 8 days (control) or in the medium supplemented with TG, CrCl_3_, MnCl_2_, FeCl_3_ or ZnSO_4_ for 4 days and then in the drug-free medium for another 4 days (treatment groups). The number of growing follicles, and the number of somatic cells with PCNA- and Ki-67-positive signals in the TG, CrCl_3_, MnCl_2_, FeCl_3_ and ZnSO_4_ treatments were significantly higher than those in the control group (Figure S4-S5). However, the number of cells with Cleaved Caspase-3-positive signals was not different between the control and treatment groups (Figure S5). Therefore, the activated follicles by the metallic compounds could grow normally.

### The metallic compounds promote the activation of mouse primordial follicles via the glycolysis-dependent mTOR pathway and the PI3K/Akt pathway

**Figure 3:**
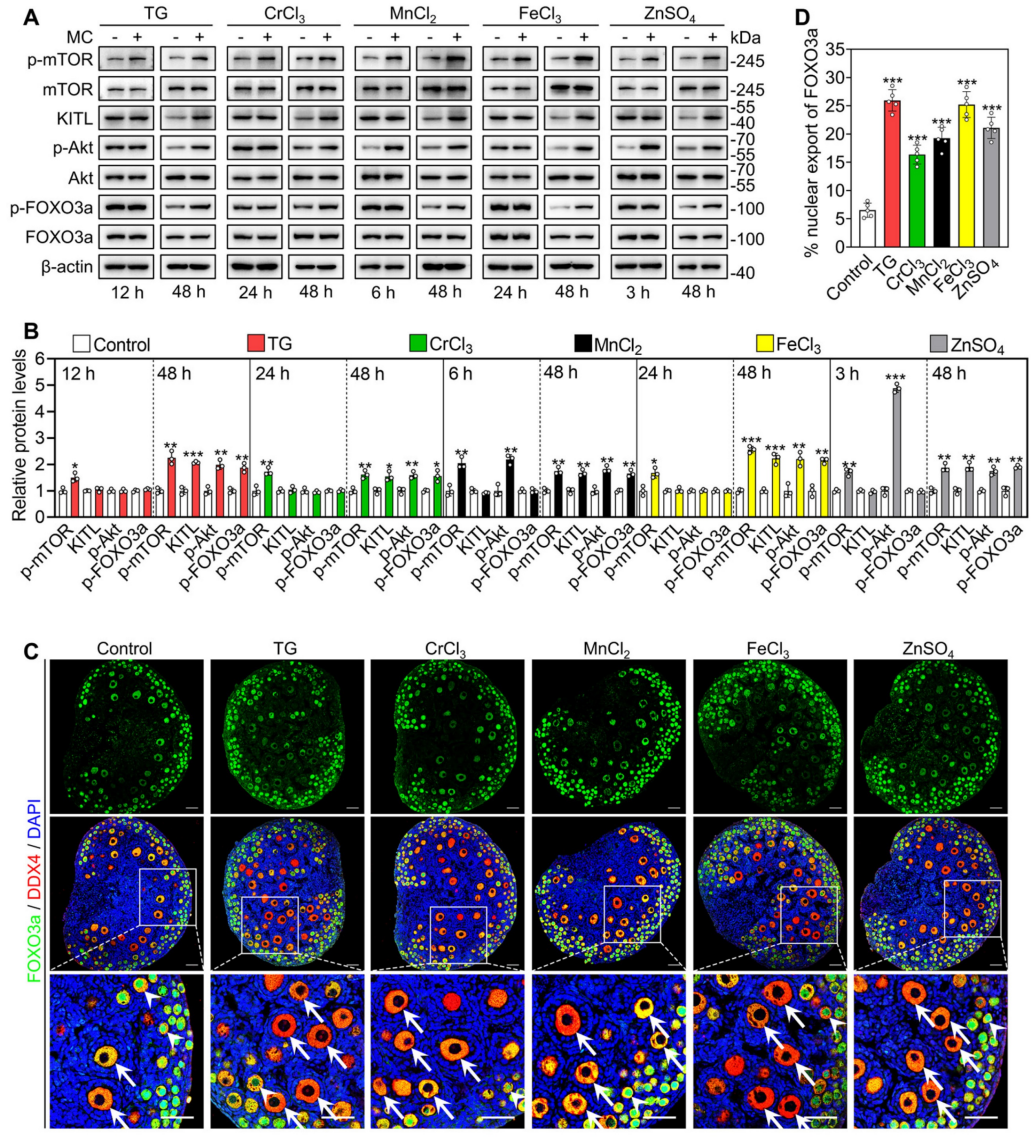
** Effects of the metallic compounds on the activation of mTOR and PI3K/Akt pathways in cultured mouse ovaries.** The ovaries from 3 dpp mice were cultured in the medium (control), or the medium supplemented with TG, CrCl_3_, MnCl_2_, FeCl_3_ or ZnSO_4_ for indicated time (**A-B**) or for 2 days (**C-D**). **A-B**, The protein levels of p-mTOR, KITL, p-Akt and p-FOXO3a in the different treatments. **C-D**, The localization of FOXO3a in oocyte cytoplasm (arrows) or nuclear (arrowheads. **C**) and the percentage of oocytes with FOXO3a nuclear export (**D**) in the different treatments. FOXO3a, green; DDX4, red; DAPI, blue. All the experiments were independently repeated three or five times, and the representative images are presented. Scale bars, 50 µm. Bars indicate the mean ± SD. *p < 0.05, **p < 0.01, and ***p < 0.001.

We cultured 3 dpp mouse ovaries to detect the effects of the metallic compounds on the protein levels of phosphorylated mTOR (p-mTOR), p-Akt, p-FOXO3a and KITL. In contrast to the control group, the treatments with TG (for 12 h), CrCl_3_ (for 24 h), MnCl_2_ (for 6 h), FeCl_3_ (for 24 h) and ZnSO_4_ (for 3 h) significantly increased p-mTOR protein levels and fluorescent signal intensities in the ovaries and granulosa cells, respectively (Figure 3A-B and S6A-B). The treatments with MnCl_2_ and ZnSO_4_ also significantly increased p-Akt protein levels and fluorescent signal intensities in the ovaries and oocytes, respectively (Figure 3A-B, S6A and S6C). After treatment for 48 h, all treatments significantly increased the protein levels of p-mTOR, KITL, p-Akt and p-FOXO3a, as well as the proportion of oocytes with FOXO3a nuclear export in contrast to the control group (Figure 3A-D).

**Figure 4:**
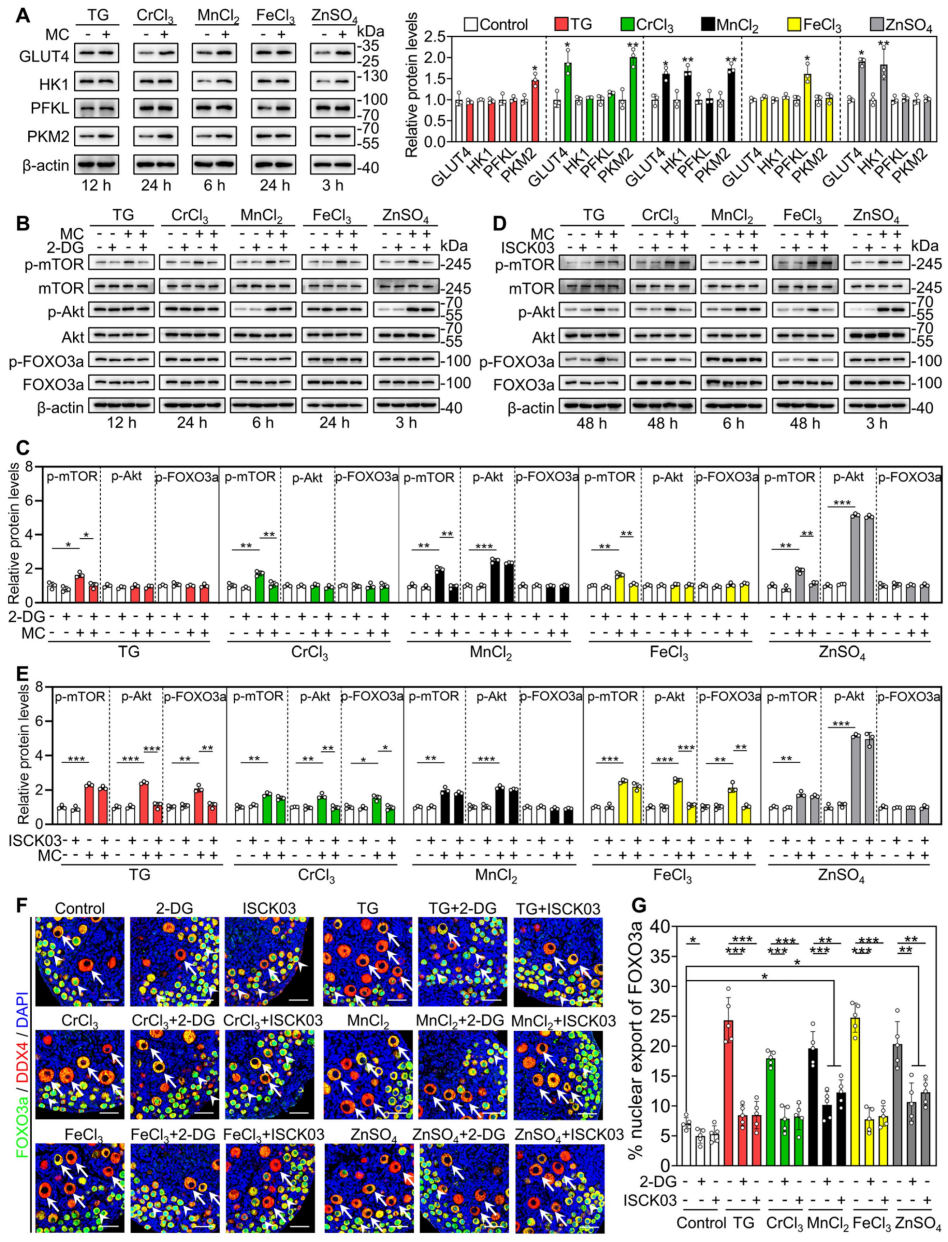
** Effects of 2-DG and ISCK03 on the metallic compound-promoted mTOR and PI3K/Akt pathways in cultured mouse ovaries.** The ovaries from 3 dpp mice were cultured in the medium (control) or the medium supplemented with TG, CrCl_3_, MnCl_2_, FeCl_3_, ZnSO_4_, 2-DG (5 mM) and/or ISCK03 (2.5 μM) for indicated time **(A-E)**, or for 2 days** (F-G)**. **A-E**, The protein levels of GLUT4, HK1, PFKL, PKM2 (**A**), and the protein levels of p-mTOR, p-Akt and p-FOXO3a (**B-E**) in the different treatments. **F-G**, The localization of FOXO3a in oocyte cytoplasm (arrows) or nuclear (arrowheads. **F**) and the percentage of oocytes with FOXO3a nuclear export (**G**) in the different treatments. FOXO3a, green; DDX4, red; DAPI, blue. All the experiments were independently repeated three or five times, and the representative images are presented. Scale bars, 50 µm. Bars indicate the mean ± SD. *p < 0.05, **p < 0.01, and ***p < 0.001.

Next, we studied the role of glycolysis in the activation of the mTOR pathway by the metallic compounds. In contrast to the control group, TG, CrCl_3_, MnCl_2_, FeCl_3_ and ZnSO_4_ significantly increased the levels of glucose transporter type 4 (GLUT4), hexokinase 1 (HK1), pyruvate kinase M2 (PKM2) and/or phosphofructokinase, liver type (PFKL) in the cultured mouse ovaries (Figure 4A). The AMP-activated protein kinase (AMPK) is the main sensor of cellular energy status and its activity is negatively correlated with glycolysis ability 35, 36. Compared with the control, 2-DG (the inhibitor of glycolysis) significantly increased p-AMPK levels in the ovaries (Figure S7), suggesting that 2-DG could inhibit glycolysis in the ovaries. TG, CrCl_3_, MnCl_2_, FeCl_3_ and ZnSO_4_ significantly decreased p-AMPK levels in the ovaries, which could be completely reversed by 2-DG (Figure S7). These results suggest that TG-, CrCl_3_-, MnCl_2_-, FeCl_3_- and ZnSO_4_-enhanced glycolysis activates mTOR pathway by inhibiting AMPK activity. 2-DG also completely blocked the compound-promoted p-mTOR levels (Figure 4B-C). 2-DG could completely block TG-, CrCl_3_- and FeCl_3_-promoted the proportion of oocytes with FOXO3a nuclear export and the number of growing follicles, and partially block MnCl_2_- and ZnSO_4_-promoted the proportion of oocytes with FOXO3a nuclear export and the number of growing follicles (Figure 4F-G and S8-S9).

Furthermore, we used ISCK03 (the KIT inhibitor, the blockade of communication network between granulosa cells and oocyte) to study the effects of the compounds on mTOR and Akt activities. Compared with the control group, ISCK03 completely blocked TG-, CrCl_3_- and FeCl_3_-promoted p-Akt and p-FOXO3a levels (Figure 4D-E). However, ISCK03, as well as 2-DG, had no effect on MnCl_2_- and ZnSO_4_-promoted p-Akt levels (Figure 4B-E). ISCK03 also completely blocked TG-, CrCl_3_- and FeCl_3_-promoted the proportion of oocytes with FOXO3a nuclear export and the number of growing follicles, and partially blocked MnCl_2_- and ZnSO_4_-promoted the proportion of oocytes with FOXO3a nuclear export and the number of growing follicles (Figure 4F-G and S8-S9). ISCK03 had no effect on KIT protein levels (Figure S10). These results indicate that these five compounds can activate the glycolysis-dependent mTOR pathway in granulosa cells, and MnCl_2_ and ZnSO_4_ can also activate the PI3K/Akt pathway in oocytes during primordial follicle activation.

**Figure 5:**
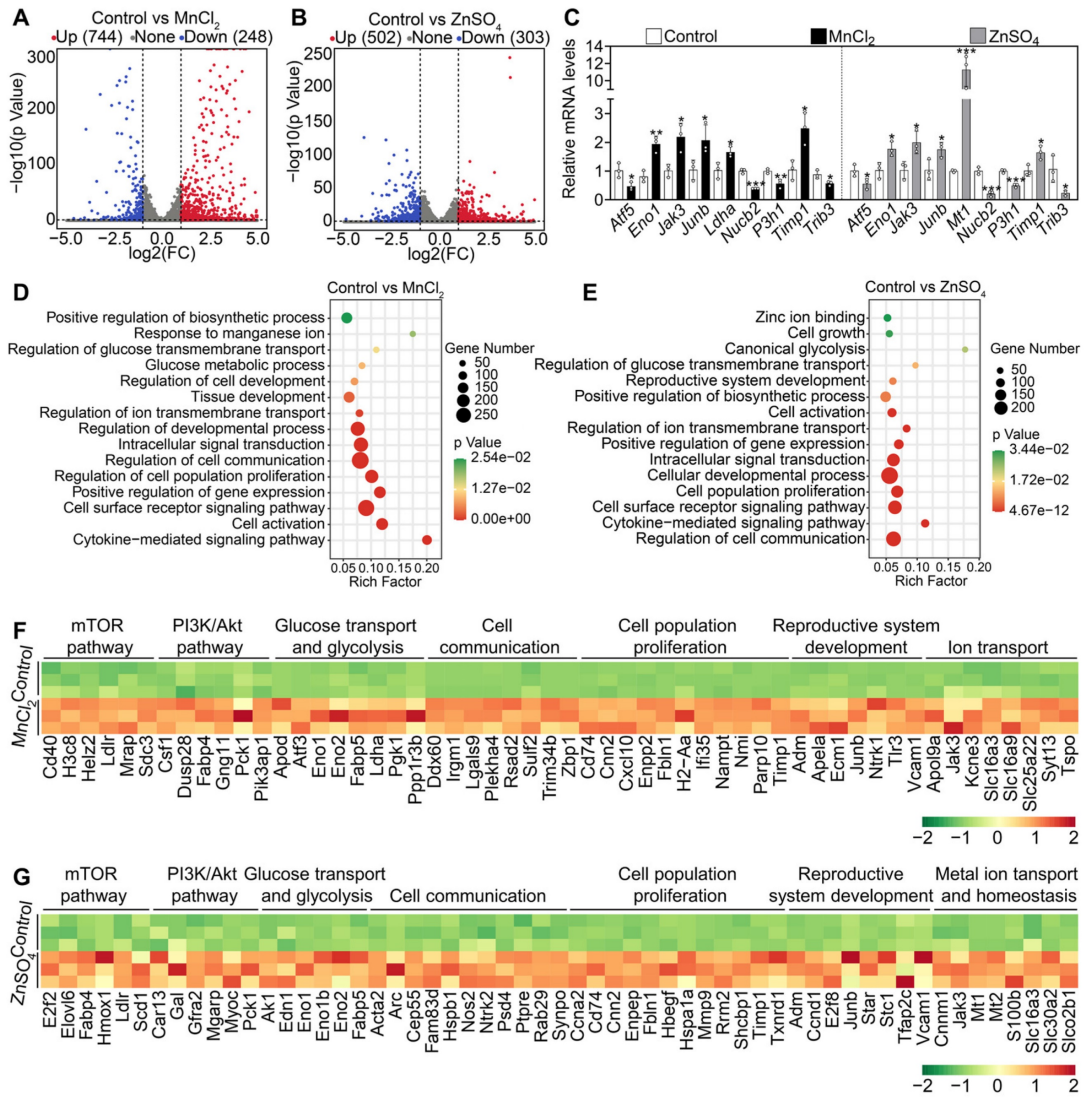
** Effects of MnCl_2_ and ZnSO_4_ on ovarian transcriptome in cultured mouse ovaries.** The ovaries from 3 dpp mice were cultured in the medium (control) or the medium supplemented with MnCl_2_ or ZnSO_4_ for 1 day. **A-B**, Volcano plot illustrating the differentially expressed genes in MnCl_2_ (**A**) and ZnSO_4_ groups (**B**). **C**, qRT-PCR validating changes in the representative transcripts selected from RNA-seq data. **D-E**, Bubble chart showing the enriched GO terms associated with the significantly upregulated transcripts in MnCl_2_ (**D**) and ZnSO_4_ groups (**E**). **F-G**, Heatmaps showing differences between control and MnCl_2_ (**F**)/ZnSO_4_ (**G**) groups in the expression of a group of transcripts involved in various processes. All the experiments were independently repeated three times. Bars indicate the mean ± SD. *p < 0.05, **p < 0.01, and ***p < 0.001.

We further analyzed the transcriptome changes in the ovaries after MnCl_2_ and ZnSO_4_ treatments. A total of 992 transcripts (including 744 upregulated and 248 downregulated transcripts) in MnCl_2_ group and 805 transcripts (including 502 upregulated and 303 downregulated transcripts) in ZnSO_4_ group were differentially expressed in contrast to the control group (Figure 5A-B). Then, the changes in the expression of representative transcripts were verified through qRT*-*PCR (Figure 5C). Gene enrichment analysis revealed that the upregulated transcripts in MnCl_2_ and ZnSO_4_ groups were the genes related to mTOR pathway, PI3K/Akt pathway, cell proliferation, development, ion transport and homeostasis, glycolysis, and cell communication (Figure 5D-G). The upregulated genes related to glycolysis, mTOR and PI3K/Akt pathways are important in primordial follicle activation 3, 4, 6. The upregulated genes related to ion transport and homeostasis may be beneficial for manganese and zinc to enter oocytes to promote Akt activity and primordial follicle activation 37-41.

### The metallic compounds promote mouse primordial follicle activation *in vivo*

**Figure 6:**
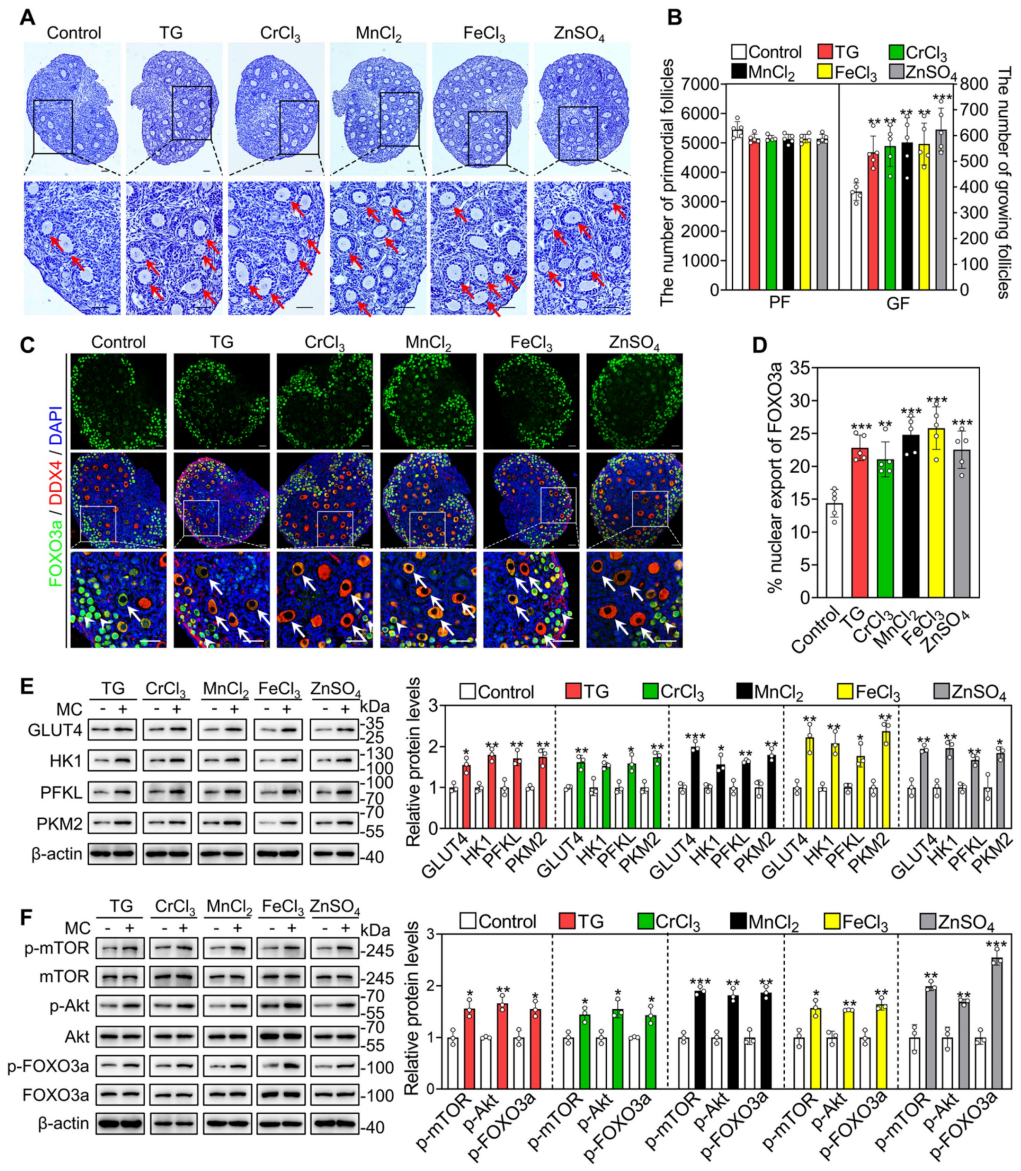
** Effects of the metallic compounds on mouse primordial follicle activation *in vivo*.** Female mice at 3 dpp were injected intraperitoneally twice a day with 0.033 mg/kg TG, 0.792 mg/kg CrCl_3_, 25.168 mg/kg MnCl_2_, 2.433 mg/kg FeCl_3_, or 7.265 mg/kg ZnSO_4_ for two consecutive days. The control mice were injected with physiological saline. The ovaries were collected after 12 h **(C-F)** and 2 days **(A-B)** of the end of injection. **A-B**, Morphological comparison of ovaries (**A**) and the number of primordial follicles (PF) and growing follicles (GF. **B**) in the different treatments. Nuclei was stained by hematoxylin. Red arrows, growing follicles. **C-D**, The localization of FOXO3a in oocyte cytoplasm (arrows) or nuclear (arrowheads. **C**) and the percentage of oocytes with FOXO3a nuclear export (**D**) in the different treatments. FOXO3a, green; DDX4, red; DAPI, blue. **E-F**, The protein levels of GLUT4, HK1, PFKL, PKM2 (**E**) and the protein levels of p-mTOR, p-Akt and p-FOXO3a (**F**) in the different treatments. All the experiments were independently repeated three or five times, and the representative images are presented. Scale bars, 50 µm. Bars indicate the mean ± SD. *p < 0.05, **p < 0.01, and ***p < 0.001.

Female mice at 3 dpp were injected intraperitoneally twice a day with different compounds for two consecutive days. Compared with the control group, the compounds significantly increased the number of growing follicles, the proportion of oocytes with FOXO3a nuclear export and the levels of glycolysis-related proteins (GLUT4, HK1, PKM2 and PFKL), p-mTOR, p-Akt and p-FOXO3a (Figure 6A-F). These results suggest that these metallic compounds promote primordial follicle activation via the glycolysis-dependent mTOR pathway and the PI3K/Akt pathway in neonatal mice.

**Figure 7:**
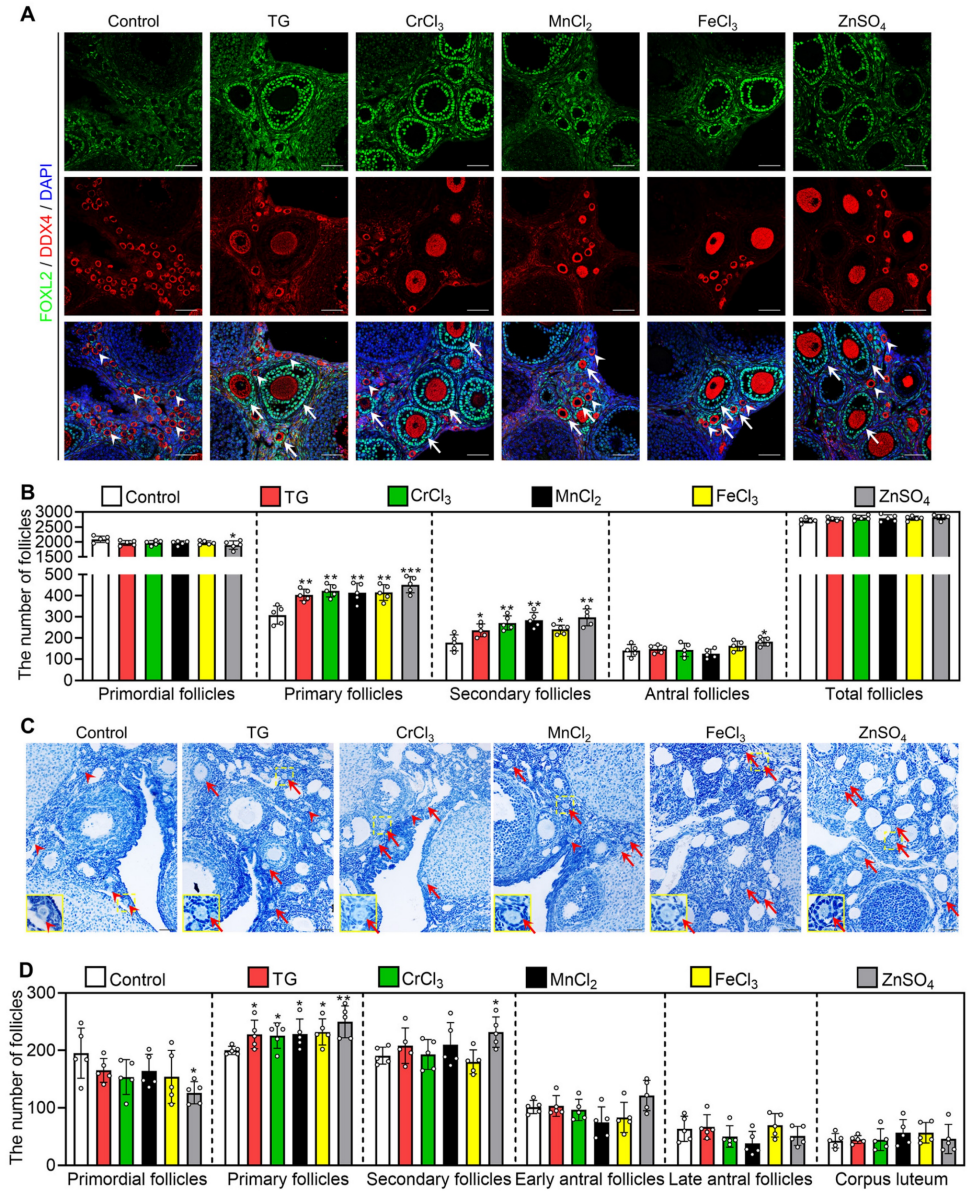
** Effects of oral administration of the metallic compounds on the activation of mouse primordial follicles.** The mice were fed normal water or the water supplemented with 0.35 μM TG, 25 μM CrCl_3_, 800 μM MnCl_2_, 65 μM FeCl_3_ or 200 μM ZnSO_4_ for one week. The ovaries were collected for follicle counting at the end of oral administration. **A-B**, Morphological comparison of ovaries (**A**) and the number of primordial and growing follicles including primary, secondary and antral follicles (**B**) in the different treatments of adolescent mice. FOXL2, green; DDX4, red; DAPI, blue. White arrowheads, primordial follicles; white arrows, growing follicles. **C-D**, Morphological comparison of ovaries (**C**) and the number of primordial, primary, secondary, early antral and late antral follicles, and corpus luteum (**D**) in the different treatment groups of aged mice. Red arrowheads, primordial follicles; red arrows, growing follicles. The small yellow boxes indicate the location of the enlarged areas, as shown in the lower left corners. All the experiments were independently repeated five times, and the representative images are presented. Scale bars, 50 µm. Bars indicate the mean ± SD. *p < 0.05, **p < 0.01, and ***p < 0.001.

Next, we investigated the effect of oral administration of these compounds on the activation of primordial follicles. In adolescent mice, the quantities ingested were 0.09-0.11 mg/kg/d TG (average 0.10 mg/kg/d), 1.60-1.68 mg/kg/d CrCl_3_ (average 1.65 mg/kg/d), 39.01-42.84 mg/kg/d MnCl_2_ (average 41.70 mg/kg/d), 3.99-4.64 mg/kg/d FeCl_3_ (average 4.35 mg/kg/d) and 13.49-14.80 mg/kg/d ZnSO_4_ (average 14.10 mg/kg/d). The oral administration of CrCl_3_, MnCl_2_, FeCl_3_ and ZnSO_4_ also significantly increased the concentration of the corresponding ions in the serum compared with the control group (Figure S11A-D). All of these compounds significantly increased the number and the proportion of primary and secondary follicles but had no effect on the number of total follicles, the morphologies of ovary, liver, spleen, kidney or small intestine, or the body weight of mice (Figure 7A-B, S11E and S12). ZnSO_4_ also increased the number of antral follicles (Figure 7A-B), consistent with a study showing that zinc can promote follicle development 42.

Furthermore, we studied the effect of these compounds on primordial follicle activation in 10-month-old female mice (aged mice), which are commonly used as a low infertility model for drug efficacy studies 43, 44. The quantities ingested were 0.07-0.09 mg/kg/d TG (average 0.08 mg/kg/d), 1.32-1.54 mg/kg/d CrCl_3_ (average 1.41 mg/kg/d), 31.51-39.88 mg/kg/d MnCl_2_ (average 35.03 mg/kg/d), 3.42-4.30 mg/kg/d FeCl_3_ (average 3.86 mg/kg/d) and 10.61-13.11 mg/kg/d ZnSO_4_ (average 11.43 mg/kg/d). Compared with the control group, these compounds significantly increased the number and the proportion of primary and/or secondary follicles, but had no effect on body weight (Figure 7C-D and S13A-B). Thus, these results suggest that the oral administration of these metallic compounds promotes primordial follicle activation in adolescent and aged mice.

### The oral administration of ZnSO_4_ rescues infertility in aged female mice

**Figure 8:**
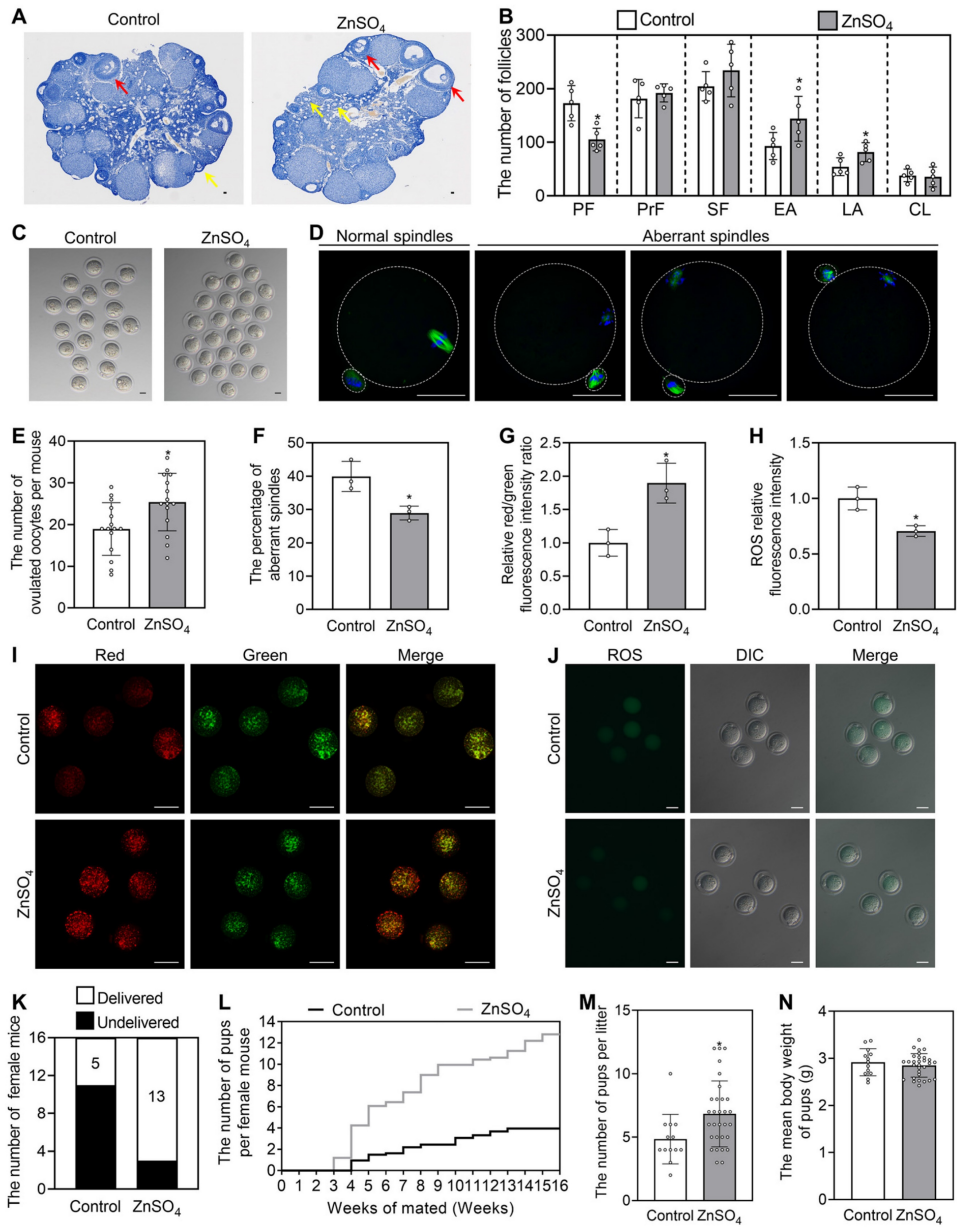
** The oral administration of ZnSO_4_ rescues infertility in aged female mice.** The aged mice were fed normal water or the water supplemented with 200 μM ZnSO_4_ for one week, and then fed normal water for further 3 weeks for follicle counting **(A-B)**, ovulated oocyte counting** (C and E)**, oocyte quality** (D and F-J)** and fertility test** (K-N)**. **A-B**, Morphological comparison of ovaries (**A**) and the number of primordial follicles (PF), primary follicles (PrF), secondary follicles (SF), early antral follicles (EA), late antral follicles (LA) and corpus luteum (CL. **B**) in the control and ZnSO_4_ groups. Nuclei was stained by hematoxylin. Yellow arrows, early antral follicles; red arrows, late antral follicles. **C and E**, Comparison of ovulated oocytes in the control and ZnSO_4_ groups. **D and F**, Morphologies of normal and aberrant spindles (**D**) and the proportion of aberrant spindles in the control and ZnSO_4_ groups (**F**). **G and I**, Relative red/green fluorescence intensity ratio (**G**) and oocyte ΔΨm shown by JC-1 staining (**I**) in the control and ZnSO_4_ groups. Red and green represent the higher and lower ΔΨm, respectively. **H and J**, The ROS relative fluorescence intensity (**H**) and the fluorescence staining of ROS (green. **J**) in the control and ZnSO_4_ groups. **K-M**, The number of aged mice with different fertility status (**K**), pups per female (**L**) and pups per litter (**M**) in the control and ZnSO_4_ groups. **N**, The mean body weight of pups per litter in the control and ZnSO_4_ groups. The representative images are presented. Scale bars, 50 µm. Bars indicate the mean ± SD. *p < 0.05.

We selected the most effective compound (ZnSO_4_) to study its effect on fertility in aged female mice. The aged mice were fed normal water or water supplemented with 200 μM ZnSO_4_ for one week, and then fed normal water for another 3 weeks for the fertility test. Compared with the control group, ZnSO_4_ increased the number of early and late antral follicles, but had no effect on weight (Figure 8A-B and S13C). ZnSO_4_ also significantly increased the number of ovulated oocytes and the oocyte mitochondrial membrane potential (ΔΨm), and significantly decreased the percentage of aberrant spindles and the content of reactive oxygen species (ROS) (Figure 8C-J). Thus, these results demonstrate that ZnSO_4_ treatment increases oocyte quantity and quality. The increase in ovulated oocytes may occur by promoting primordial follicle activation.

In the three-month mating trials (n = 16 in each group), 5 and 13 mice delivered pups in the control and ZnSO_4_ groups, respectively (Figure 8K). Compared with the control, ZnSO_4_ notably increased the average number of pups per mouse and significantly increased the number of pups per litter (Figure 8L-M). 13 litters of mice were born in control and 30 litters of mice were born in ZnSO_4_ group. The newborn mice had no obvious malformation, and the weight was not different between the ZnSO_4_ group and the control group (Figure 8N). These results demonstrate that the oral administration of ZnSO_4_ is able to enhance fertility by increasing oocyte quantity and quality in aged female mice.

### The metallic compounds promote human primordial follicle activation *in vitro*

**Figure 9:**
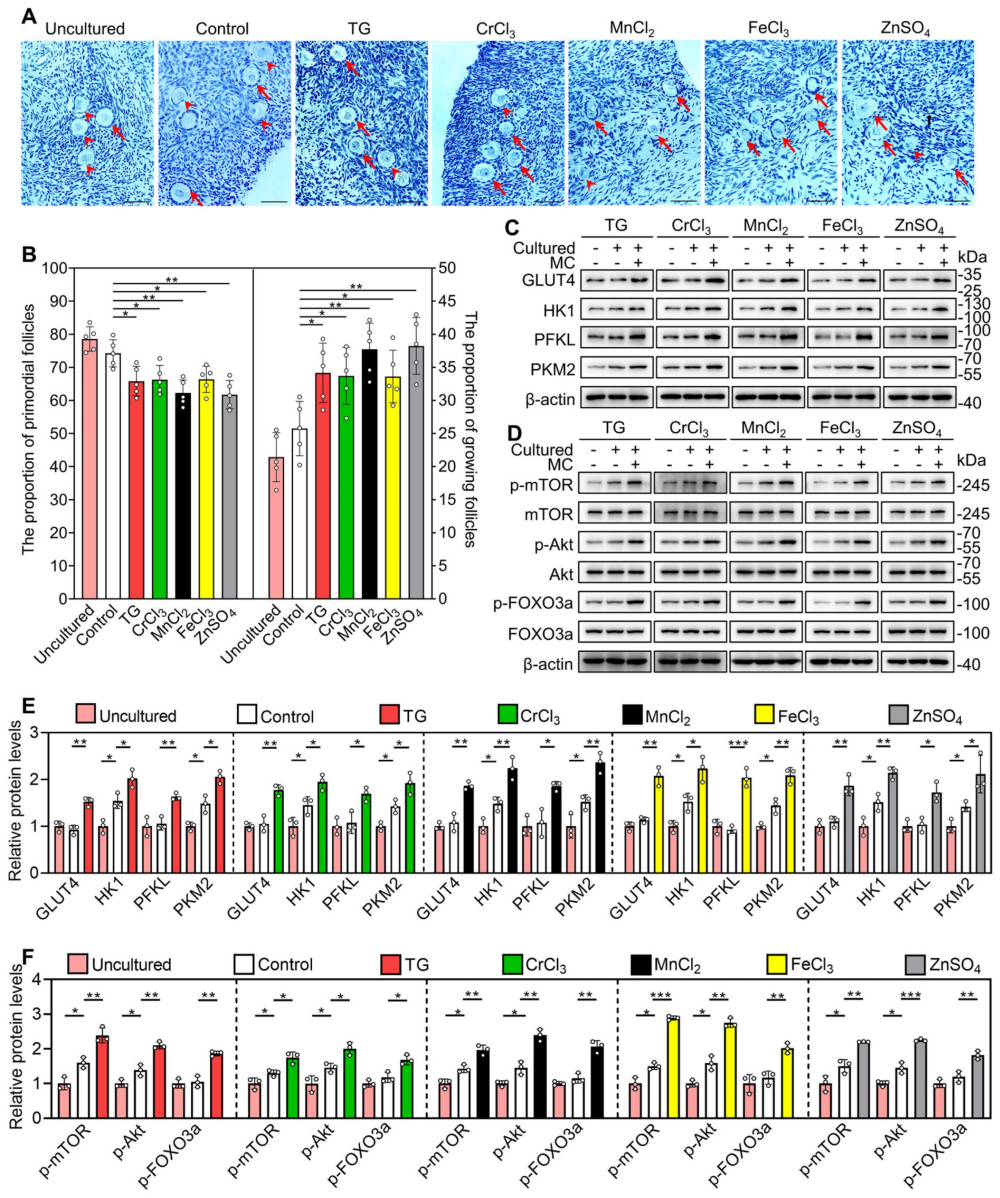
** Effects of the metallic compounds on human primordial follicle activation *in vitro*.** Human ovarian fragments were cultured in the medium (control), or the medium supplemented with TG, CrCl_3_, MnCl_2_, FeCl_3_ or ZnSO_4_ for 4 days and then in the drug-free medium for another 2 days. The fragments were collected at the end of 4-day culture **(C-F)** or 6-day culture** (A-B)**. For uncultured group, human ovarian fragments were collected for histological analysis and protein detection. **A-B**, Morphological comparison of human ovarian tissue fragments (**A**) and the proportion of primordial and growing follicles (**B**) in the different treatments. Nuclei were stained by hematoxylin. Arrowheads, primordial follicles; arrows, growing follicles. **C-F**, The protein levels of GLUT4, HK1, PFKL, PKM2 (**C and E**) and the protein levels of p-mTOR, p-Akt and p-FOXO3a (**D and F**) in the different treatments. All the experiments were independently repeated three or five times, and the representative images are presented. Scale bars, 50 µm. Bars indicate the mean ± SD. *p < 0.05, **p < 0.01, and ***p < 0.001.

We further explored the effects of these compounds on the activation of primordial follicles in human ovary tissues. The ovary fragments were cultured in the medium (control) or the medium supplemented with different drugs for 4 days for protein analysis or for 6 days for follicle count. Compared with the control group, TG, CrCl_3_, MnCl_2_, FeCl_3_ and ZnSO_4_ significantly increased the proportion of growing follicles and the levels of glycolysis-related proteins (GLUT4, HK1, PKM2 and PFKL), p-mTOR, p-Akt and p-FOXO3a (Figure 9A-F). These results suggest that these compounds promote human primordial follicle activation via the glycolysis-dependent mTOR pathway and the PI3K/Akt pathway. Otherwise, in contrast to the uncultured group, the proportion of growing follicles was slightly increased, and the levels of HK1, PKM2, p-mTOR and p-Akt were significantly increased (Figure 9A-F), suggesting the initiation of follicle growth in the control group 45, 46.

## Discussion

In mammals, trace elements are required and crucial for reproduction. In this study, we found that 11 metallic compounds, including AlCl_3_, TG, CrCl_3_, MnCl_2_, FeCl_3_, ZnSO_4_, NaAsO_2_, Na_2_SeO_3_, Na_2_MoO_4_, GdCl_3_ and Pb(CH_3_CO_2_)_2_, activated mouse primordial follicles. Further study indicated that TG, CrCl_3_, MnCl_2_, FeCl_3_ and ZnSO_4_ activated mouse and human primordial follicles by the glycolysis-dependent mTOR pathway and/or the PI3K/Akt pathway. The oral administration of ZnSO_4_ also promoted primordial follicle activation, improved oocyte quality and rescued infertility in aged mice.

The activation of mTOR by enhancing glycolysis in pregranulosa cells and the PI3K/Akt pathway in oocytes play critical roles in primordial follicle activation 2, 4, 6. It has been reported that calcium, trivalent chromium, manganese, zinc and iron can stimulate glycolysis by increasing glycolysis-associated gene and protein levels, and promoting glucose uptake and enzyme activity in various tissues 24, 26, 27, 29, 47. In the present study, TG, CrCl_3_, MnCl_2_, FeCl_3_ and ZnSO_4_ increased glycolysis-associated protein levels to promote glucose metabolism in ovarian tissues, and then promoted primordial follicle activation via the glycolysis-dependent mTOR pathway in granulosa cells. Whether these compounds can also promote glycolysis via glucose uptake and enzyme activity needs further research.

Interestingly, we found that MnCl_2_ and ZnSO_4_ could also activate primordial follicles via the PI3K/Akt pathway in oocytes. Manganese can be transported into oocytes through Ca_V_3.2, transient receptor potential cation channel subfamily M member 7 (TRPM7) and transient receptor potential cation channel subfamily V member 3 (TRPV3) channels 40, and then promote superoxide dismutase (SOD) activity by forming the manganese-SOD (Mn-SOD) complex 48. Zinc can be transported into oocytes through the zinc transporter solute carrier family 39 41. We also found that the ion transport-related genes were increased in MnCl_2_ and ZnSO_4_ groups, which may be beneficial for manganese and zinc to enter oocytes to promote Akt activity and primordial follicle activation. Furthermore, Mn-SOD and zinc are reported to cause the phosphatase and tensin homolog (PTEN) inhibition and/or degradation, resulting in an increase in Akt activity in uterine leiomyomas and human airway epithelial cells, respectively 49, 50. Thus, manganese and zinc may enter oocytes to activate the PI3K/Akt pathway by inhibiting PTEN. It has also been reported that aluminum, arsenic, selenium, molybdenum, gadolinium and lead can enhance glucose metabolism, activate the mTOR pathway and/or increase Akt activity in many tissues 30, 51-55. Thus, AlCl_3_, NaAsO_2_, Na_2_SeO_3_, Na_2_MoO_4_, GdCl_3_ and Pb(CH_3_CO_2_)_2_ promote the activation of primordial follicles possibly via the mTOR pathway and/or the PI3K/Akt pathway.

Aged women and POI patients have a few residual dormant primordial follicles, and traditional ART has difficulty rescuing their infertility 11, 13. Only a few young patients have obtained their own offspring via IVA, accompanied by the risk of carcinogenesis and offspring defects 14. Here, we showed that the oral administration of the metallic compounds could promote the activation of mouse primordial follicles and had no obvious toxicity or side effect in mice. Moreover, the oral administration of ZnSO_4_ rescued infertility in aged mice by improving oocyte quantity and quality. The concentrations of TG, CrCl_3_, MnCl_2_, FeCl_3_ and ZnSO_4_ in aged mice were 0.08 mg/kg/d, 1.41 mg/kg/d (equal to 0.47 mg/kg/d chromium), 35.03 mg/kg/d (equal to 15.28 mg/kg/d manganese), 3.86 mg/kg/d (equal to 1.33 mg/kg/d iron) and 11.43 mg/kg/d (equal to 4.63 mg/kg/d zinc), respectively. TG at 1.5 mg/kg/d and chromium at 1 mg/kg/d have been used in mice to treat tumors and type II diabetes 56, 57, respectively. Iron at 6 mg/kg/d and zinc at 6 mg/kg/d have been used in humans to treat anemia and angina pectoris, respectively 58, 59. Importantly, these metallic compounds also promote the activation of human primordial follicles. Thus, TG, CrCl_3_, FeCl_3_ and ZnSO_4_, especially ZnSO_4_, could be used as safe oral drug candidates for the treatment of aged women and POI patients. Manganese at a concentration of 6.50 mg/kg/d was used to treat patients with SLC39A8 deficiency 60. Whether 20.90 mg/kg/d manganese is safe for humans needs further study. Manganese, zinc and selenium can improve oocyte quality and embryo development 61, 62, which is related to glycolysis 63. Thus, the metallic compounds may improve oocyte quality by enhancing glycolysis.

In summary, we found that the metallic compounds activated mouse and human primordial follicles via the glycolysis-dependent mTOR pathway and/or the PI3K/Akt pathway. The oral administration of ZnSO_4_ also rescued infertility in aged mice. Because of their efficacy, safety and practicability, these metallic compounds may be provided as a new clinical approach toward rescuing infertility in aged women and POI patients.

## Materials and methods

### Experimental animals and chemicals

All ICR (CD1) mice at the ages of 21 days postpartum (dpp), 2 and 10 months were purchased from the Guangdong Medical Laboratory Animal Center (Guangzhou, China) and kept in the animal facility with a 12/12 h light/dark cycle, with freely available water and food under the controlled temperature of 22 ± 2 °C and 50-70% humidity at South China University of Technology. Two-month-old male and female mice were mated at a ratio of 1:1 to generate newborn mice. The day of birth was considered to be 0.5 dpp. Female mice at 3 dpp were used for ovary culture or for intraperitoneal injection with different drugs. For the oral administration experiment, 21-day-old and 10-month-old mice were fed normal water or water supplemented with different drugs. The reagents in this study, unless stated otherwise, were purchased from Sigma‒Aldrich (St. Louis, MO, USA).

### Mouse ovary culture

The ovaries were separated from 3 dpp female mice in sterile phosphate buffered saline (PBS) and were cultured on a Millipore insert (PICMORG50, Millipore, Billerica, MA, USA) in a six-well culture plate (NEST, Beijing, China). Each well contained 3 mL of Dulbecco's modified Eagle's medium/Ham's F12 nutrient mixture (Thermo Fisher Scientific, Waltham, MA, USA) supplemented with 3 mg/mL bovine serum albumin, 1% insulin-transferrin-selenium (ITS) and 100 UI/mL penicillin-streptomycin. The ovaries were cultured in medium supplemented with AlCl_3_ (0-10 μM), TG (0-0.1 μM), CrCl_3_ (0-25 μM), C_8_H_4_K_2_O_12_Sb_2_ (0-25 μM), MnCl_2_ (0-250 μM), FeCl_3_ (0-20 μM), CoCl_2_ (0-150 μM), NiCl_2_ (0-150 μM), CuCl_2_ (0-50 μM), ZnSO_4_ (0-55 μM), NaAsO_2_ (0-5 μM), Na_2_SeO_3_ (0-4 μM), Na_2_MoO_4_ (0-100 μM), CdCl_2_ (0-1 μM), KIO_3_ (0-150 μM), GdCl_3_ (0-20 μM), Pb(CH_3_CO_2_)_2_ (0-50 μM), 2-DG (glycolysis inhibitor, 5 mM) and/or ISCK03 (KIT inhibitor, 2.5 μM). TG, 2-DG and ISCK03 were prepared as stock solutions in dimethyl sulfoxide (DMSO), and the others were prepared as stock solutions in ultrapure water. They were diluted with culture medium before use. The concentration of DMSO is no more than 0.1% in the cultured system, and the same concentration of DMSO was also added to the corresponding control group. All cultures were carried out at 37 °C with 5% CO_2_ and saturated humidity, and the medium was changed every 2 days. The cultured ovaries were collected at the designated times for follicle counting, immunofluorescence staining, and gene and protein detection.

### Mouse injection and oral administration experiment

The single injection dose of drugs (mg/kg) was the same as the most effective concentration *in vitro*, in which the volume ratio (mg/L) was replaced by the mass ratio (mg/kg). Female mice at 3 dpp were injected intraperitoneally twice a day with 0.033 mg/kg TG, 0.792 mg/kg CrCl_3_, 25.168 mg/kg MnCl_2_, 2.433 mg/kg FeCl_3_, or 7.265 mg/kg ZnSO_4_ for two consecutive days. The total volume of each injection was 4 µL per mouse. The control mice were injected with 4 µL physiological saline. The ovaries were collected for immunofluorescence staining and protein detection 12 h after the end of injection or for follicle counting 2 days after the end of injection.

The drug concentration in drinking water was calculated based on the daily water consumption of mouse (~ 0.4 L/kg/d from the pre-experiment), in which the daily intake dose of the drug from water is the same as the daily injection dose of the drug. For the oral administration experiment, 21-day-old and aged female mice were fed normal water or water supplemented with 0.35 μM TG, 25 μM CrCl_3_, 800 μM MnCl_2_, 65 μM FeCl_3_, or 200 μM ZnSO_4_ for one week. The weight and water consumption of mice were recorded every day. The daily concentration of drugs was calculated by the quantity of ingestion (mg)/body weight (kg). The ovaries and serum were collected at the end of feeding for follicle counting and ion concentration level analysis, respectively. In some experiments, the aged mice were further fed normal water for 3 weeks for follicle counting, oocyte quality analysis and fertility testing.

### Measurement of ion concentration levels

The serum was collected from adolescent mice after different treatments. 30 μL serum and 1470 μL ultrapure water were mixed in the 2 mL tube. The concentrations of ions were determined by inductively coupled plasma-mass spectrometry (ICP-MS 7850, Agilent, Palo Alto, CA, USA). The specific mass-to-charge ratio (m/z) is used to determine the type of ions. Quantitative analysis was carried out using internal standard method.

### Fertility test and ovulation analysis

For the fertility test, aged mice with different treatments were mated with fertile adult male mice for 3 months. The number of newborn mice was counted once a week to plot a reproductive curve. For ovulation analysis, the mice were intraperitoneally injected with 5 IU equine chorionic gonadotropin 48 h later followed by 5 IU human chorionic gonadotrophin. After 14 h, the oocytes were collected by removing cumulus cells with 0.1% hyaluronidase. The number of oocytes was recorded, and the quality of oocytes was detected by spindle staining, ROS and ΔΨm measurement.

### ROS and ΔΨm measurement

The measurement of ROS and ΔΨm in oocytes was conducted according to the instructions (Beyotime). For ROS detection, the oocytes were stained in M2 medium with 10 μM 2',7'-dichlorodihydrofluorescein diacetate and then washed three times in M2 medium. For ΔΨm detection, the oocytes were stained in buffer solution with JC-1 and then washed three times in buffer solution. The oocytes were then placed on glass bottom cell culture dishes (NEST, Beijing, China) and photographed by confocal microscopy (LSM 800, Carl Zeiss, Oberkochen, Germany).

### Human ovary tissue culture

Fourteen women aged 23-41 years (31.3 ± 6.5 years) donated small ovary cortical biopsy specimens (adjacent nonpathological tissue) while undergoing routine gynecological laparoscopies at Guangzhou First People's Hospital, Guangzhou, Guangdong, China. We obtained informed consent before surgery, and the study was conducted in accordance with the Declaration of Helsinki. The collection and use of human ovarian tissue were approved by the ethics committee of Guangzhou First People's Hospital (No. K-2021-184-01). The ovary tissues were immediately transported to the laboratory within cold preequilibrated Dulbecco's phosphate-buffered saline with ITS and penicillin-streptomycin.

The ovary tissues were divided into fragments of ~1 mm^3^ in sterile conditions. Some fragments from each tissue were collected for protein detection or were fixed in 4% paraformaldehyde (PFA, Solarbio, Beijing, China) for serial sections and then stained with hematoxylin for follicle counting (uncultured group). The other fragments were divided equally and randomly for culture in medium (control) supplemented with 0.05 μM TG, 5 μM CrCl_3_, 200 μM MnCl_2_, 15 μM FeCl_3_, or 35 μM ZnSO_4_ for 4 days and then in drug-free medium for another 2 days. The fragments were collected to analyze the protein levels after 4 days of culture or to count the follicle number after 6 days of culture.

### Histological and morphological analysis

The ovary samples were fixed in 4% PFA overnight and then embedded in paraffin (Leica Biosystems, Wetzlar, Germany). The paraffins were serially sectioned at 5 µm thickness, and these sections were deparaffinized, hydrated and stained with hematoxylin (Solarbio). In neonatal mouse ovaries, the primordial follicles in every fifth section were counted and the number of total primordial follicles in each ovary was calculated based on the formula (average follicle number per section × total section numbers). The growing and atretic follicles were counted in serial sections. In other mouse ovaries and human ovary tissues, the primordial follicles and growing follicles, including primary follicles, secondary follicles and antral follicles, were counted in serial sections. The follicles with a clearly visible oocyte nucleus were counted. Each follicle was counted only once. All sections were counted by two independent individuals for comparison.

### Immunofluorescent staining

The ovary sections were subjected to antigen retrieval using sodium citrate buffer (pH 6.0) at 95-98 ℃. Then, the sections were cooled for 2-4 h and blocked with 10% donkey serum (Solarbio) at room temperature for 60 minutes. After incubation with primary antibodies (Supplementary Table S1) at 4 °C overnight, these sections were incubated with Alexa Fluor 488- or 555-conjugated secondary antibodies (1:200. Thermo Fisher Scientific) at room temperature for 2-3 h. These sections were then washed with PBS, stained with 4′,6-diamidino-2-phenylindole (DAPI, 1:200. Solarbio) for 3 min and covered with anti-fluorescence quenching agents (Ruitaibio, Beijing, China). The sections were photographed by confocal microscopy (LSM 800, Carl Zeiss). The five largest sections in each ovary were used to obtain the proportion of granulosa cells with positive signals and the proportion of oocytes with FOXO3a nuclear export. The proportion of granulosa cells with positive signals was calculated based on the formula (the number of granulosa cells with positive signals/the total number of granulosa cells in one section). The proportion of oocytes with FOXO3a nuclear export was calculated based on the formula (the number of oocytes with FOXO3a nuclear export/the total number of oocytes in one section). The mean proportion of five sections was considered the data of one independent sample. All counts were completed by two independent individuals for comparison. The fluorescence intensity was analyzed using Zeiss Zen 3.0 software (Carl Zeiss). The relative fluorescence intensity was calculated by dividing the fluorescence intensity of cells by the fluorescence intensity of the background.

Oocytes from aged mice subjected to different treatments were fixed in 4% PFA at room temperature for 30 min and then permeabilized for 20 min in PBS containing 0.5% Triton X-100. After blocking for 1 h in 10% donkey serum at 37 °C, the oocytes were incubated with anti-alpha tubulin antibody conjugated with Alexa Fluor 488 (1:300) for 3 h at 37 °C. After washing with PBS containing 0.5% Tween, the oocytes were stained with DAPI for 15 min. The oocytes were then placed on slides with anti-fluorescence quenching agents and photographed by confocal microscopy.

### Western blotting analysis

Total protein from ovaries in each treatment was extracted using radio immunoprecipitation assay lysis buffer (Beyotime, Beijing, China) with 1 mM phenylmethylsulfonyl fluoride (Beyotime). Then, the quantity of protein was detected by bicinchoninic acid assay (Beyotime). Fifteen micrograms of protein from each treatment mixed with sodium dodecyl sulfate (SDS, Cwbio, Beijing, China) buffer were separated using 10% SDS-polyacrylamide gels by electrophoresis and subsequently transferred to pure polyvinylidene fluoride (PVDF) membranes (Millipore). After blocking nonspecific binding at room temperature for 1 h with 5% skim milk (Absin, Shanghai, China), the membranes were incubated with primary antibodies (Supplementary Table S1) overnight at 4 °C. The membranes were washed with Tris-buffered saline with Tween 20 and incubated with anti-mouse or anti-rabbit IgG secondary antibody (1:5000. ZSGB-BIO, Beijing, China) at room temperature for 1 h. Finally, the protein bands in the membranes were visualized by SuperSignal West Pico Chemiluminescent Substrate (Thermo Fisher Scientific) and photographed by the Tanon 5200 chemiluminescent imaging system (Tanon, Shanghai, China). The band density was analyzed by ImageJ software (NIH Image, Bethesda, MD, USA), and the protein levels were normalized to those of β-actin. All uncropped blots are shown in Supplementary Figure S14-S16.

### RNA extraction and quantitative real-time PCR

Total RNA of eight mouse ovaries in each treatment was isolated using TRIzol reagent (Thermo Fisher Scientific), and the quality and concentration of RNA were detected by a NanoDrop™ One Spectrophotometer (Thermo Fisher Scientific). One microgram of total RNA from each treatment was reverse transcribed into cDNA using the GoScript™ Reverse Transcription System (Promega, Madison, WI, USA). Quantitative real-time PCR (qRT‒PCR) was carried out using TransStart® Tip Green qPCR SuperMix (Trans, Beijing, China) on a Light Cycler 96 instrument (Roche, Basel, Switzerland). The quantification of mRNAs was calculated using the ribosomal protein L19 (*Rpl19*) signal as the internal control. The primers were synthesized by BGI Genomics (BGI-Tech, Shenzhen, China), and the sequences are listed in Supplementary Table S2.

### RNA-Sequencing analysis

The ovaries from 3 dpp mice were cultured in the medium (control) or the medium supplemented with 200 μM MnCl_2_ or 35 μM ZnSO_4_ for 1 day. The ovaries were collected to extract total RNA for RNA-sequencing assay. The cDNA library sequencing was sequenced using Illumina Novaseq6000 by Gene Denovo Biotechnology Co., Ltd (Guangzhou, China). The analysis was performed by R and DESeq2 software. The images were graphed using Omicstudio (www.omicstudio.cn).

### Bromodeoxyuridine (BrdU) incorporation assay

The ovaries from 3 dpp female mice were cultured for 2 days with different drugs and then cultured in drug-free medium supplemented with 10 μM BrdU for 2 h. At the end of culture, these ovaries were collected for serial section as described above. After retrieval and blocking, the sections were incubated with anti-BrdU antibody overnight at 4 ℃ and then incubated with Alexa Fluor 488-conjugated donkey secondary antibody at room temperature for 2-3 h. The largest five sections in each ovary were used to count the number of somatic cells with positive signals. The mean number of five sections was considered the data of one independent sample. All sections were counted by two independent individuals for comparison.

### Statistical analysis

The results are shown as the mean ± SD. The data were analyzed by two-tailed unpaired Student's t tests, and the images were graphed using GraphPad Prism software (La Jolla, CA, USA).

## Supplementary Material

Supplementary figures and tables.

## Abbreviations

Akt: protein kinase B
AMPK: AMP-activated protein kinase
ART: assisted reproductive technology
Bax: B-cell lymphoma 2-associated X
Bcl-2: B-cell lymphoma 2
BrdU: bromodeoxyuridine
CL: corpus luteum
DAPI: 4′,6-diamidino-2-phenylindole
DDX4: DEAD-box helicase 4
DMSO: dimethyl sulfoxide
EA: early antral follicles
FOXO3a: forkhead box O3a
GC: granulosa cells
Gdf9: growth differentiation factor 9
GF: growing follicles
GLUT4: glucose transporter type 4
HDAC6: histone deacetylase 6
HK1: hexokinase 1
ICP-MS: inductively coupled plasma-mass spectrometry
ITS: insulin-transferrin-selenium
IVA: *in vitro* activation
KIT: proto-oncogenic receptor tyrosine kinase
KITL: proto-oncogenic receptor tyrosine kinase ligand
LA: late antral follicles
MAPK: mitogen-activated protein kinase
MC: metallic compounds
mTOR: mammalian target of rapamycin
OO: oocytes
PBS: phosphate buffered saline
PCNA: proliferating cell nuclear antigen
PCOS: polycystic ovary syndrome
PF: primordial follicles
PFA: paraformaldehyde
PFKL: phosphofructokinase, liver type
PI3K: phosphoinositide 3-kinase
PKM2: pyruvate kinase M2
POI: premature ovarian insufficiency
PrF: primary follicles
PTEN: phosphatase and tensin homolog
qRT‒PCR: quantitative real-time PCR
ROS: reactive oxygen species
Rpl19: ribosomal protein L19
SDS: sodium dodecyl sulfate
SF: secondary follicles
SOD: superoxide dismutase
TG: thapsigargin
TRPM7: transient receptor potential cation channel subfamily M member 7
TRPV3: transient receptor potential cation channel subfamily V member 3
Zp3: zona pellucida glycoprotein 3
ΔΨm: mitochondrial membrane potential
