# Supplementary material for: The metallic compound promotes primordial follicle activation and ameliorates fertility deficits in aged mice
Source: Theranostics. 2023 May 21;13(10):3131–48. doi: 10.7150/thno.82553 (PMC10283063; doi:10.7150/thno.82553)
Supplement: Supplementary file 1 — Supplementary figures and tables. [file thnov13p3131s1.pdf]

Supporting information

The metallic compound promotes primordial follicle activation and ameliorates fertility deficits in aged mice

Lincheng Han[1#], Yingying Huang[1#], Biao Li[1], Weiyong Wang[1], Yan-li Sun[1], Xiaodan Zhang[1], Wenbo Zhang[1], Shuang Liu[1], Wenjun Zhou[1], Wei Xia[2*] and Meijia Zhang[1*]

[1]Division of Cell, Developmental and Integrative Biology, School of Medicine, South China University of Technology, Guangzhou 510006, China.

[2]Department of Reproductive Medicine Centre, Guangzhou First People's Hospital, South China University of Technology, Guangzhou, Guangdong, China 510180, China.

[#]These authors contributed equally: Lincheng Han, Yingying Huang.

*Corresponding author: Wei Xia, E-mail: rose-xw@163.com; Meijia Zhang, E-mail: zhangmeijia@scut.edu.cn.

Supplementary Figures

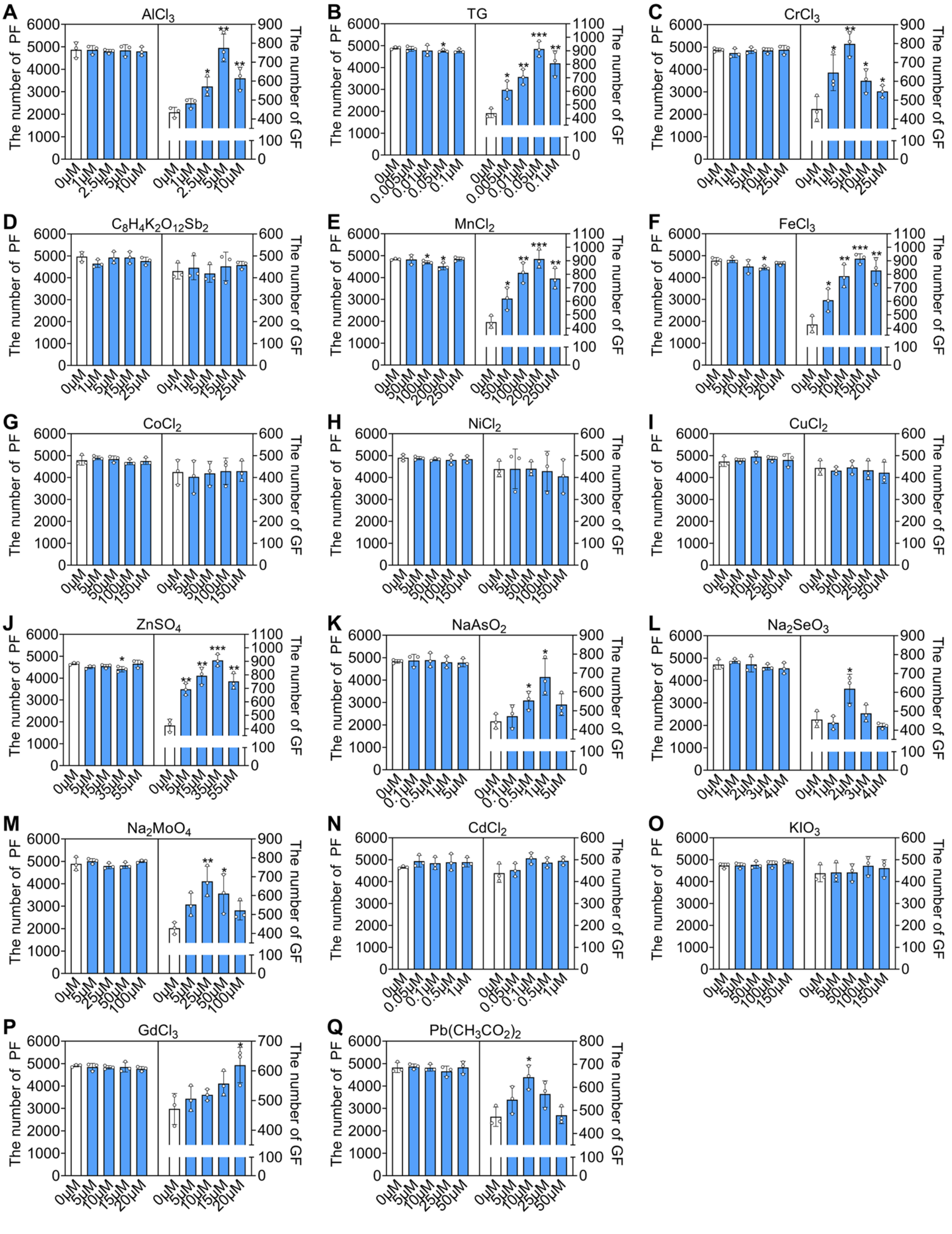

Figure S1. Effects of metallic compounds with different concentrations on the activation of primordial follicles in cultured mouse ovaries. The ovaries from 3 dpp mice were cultured in the medium (control) or the medium supplemented with $AlCl_3$, TG, $CrCl_3$, $C_8H_4K_2O_{12}Sb_2$, $MnCl_2$, $FeCl_3$, $CoCl_2$, $NiCl_2$, $CuCl_2$, $ZnSO_4$,

$NaAsO_2$, Na_2SeO_3, Na_2MoO_4, $CdCl_2$, KIO_3, $GdCl_3$ or $Pb(CH_3CO_2)_2$ with indicated concentrations for 4 days. **A–Q**, The number of primordial follicles (PF) and growing follicles (GF) in the different treatments. All the experiments were independently repeated three times. Bars indicate the mean ± SD. *$p < 0.05$, **$p < 0.01$, and ***$p < 0.001$.

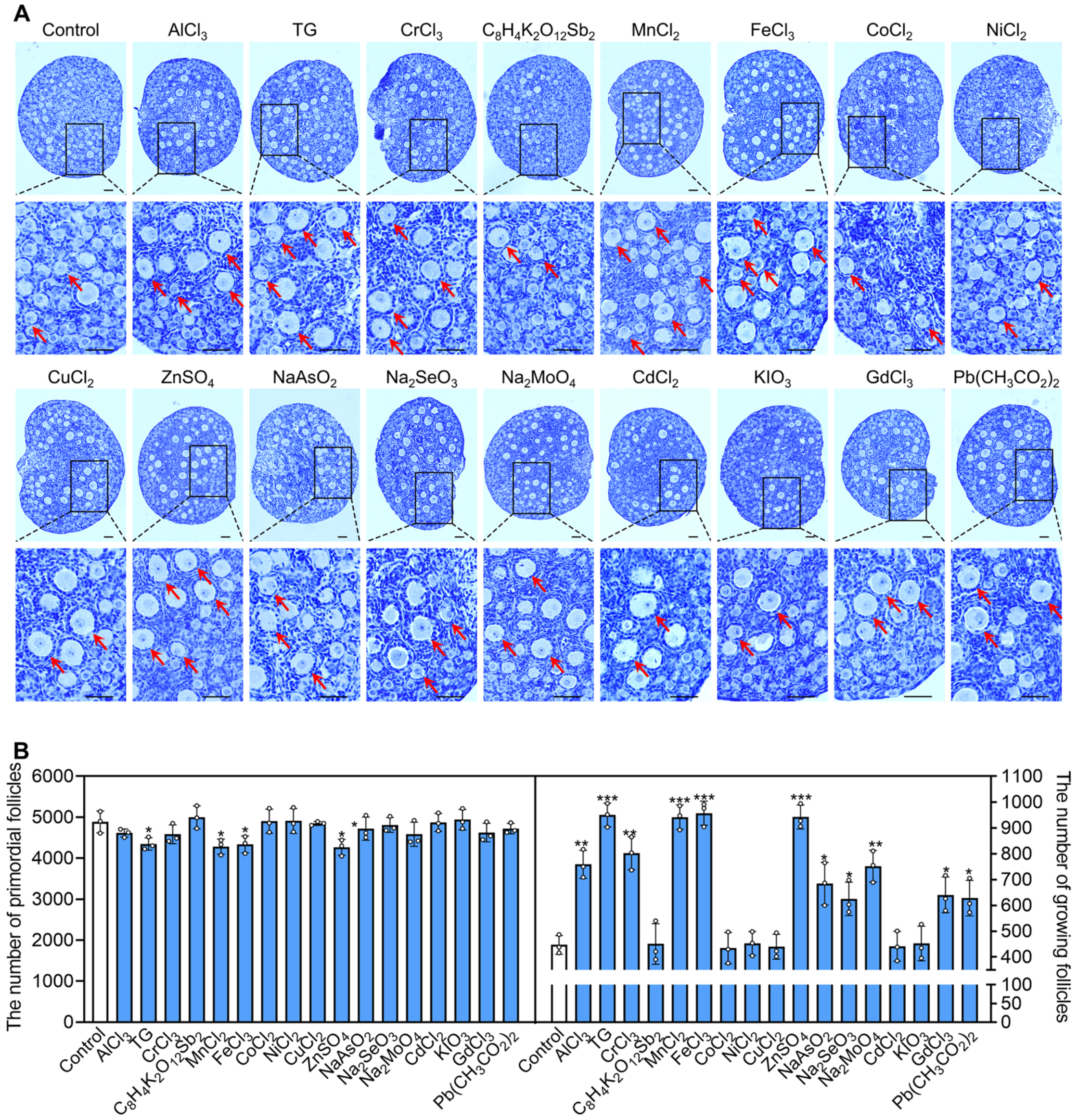

Figure S2. Effects of metallic compounds on the activation of primordial follicles in cultured mouse ovaries. The ovaries from 3 dpp mice were cultured in the medium (control), supplemented with 5 μM $AlCl_3$, 0.05 μM TG, 5 μM $CrCl_3$, 25 μM $C_8H_4K_2O_{12}Sb_2$, 200 μM $MnCl_2$, 15 μM $FeCl_3$, 150 μM $CoCl_2$, 150 μM $NiCl_2$, 50 μM $CuCl_2$, 35 μM $ZnSO_4$, 1 μM $NaAsO_2$, 2 μM Na_2SeO_3, 25 μM Na_2MoO_4, 1 μM $CdCl_2$, 150 μM KIO_3, 20 μM $GdCl_3$ or 10 μM $Pb(CH_3CO_2)_2$ for 4 days. **A–B**, Morphological comparison of ovaries (**A**) and the number of primordial and growing follicles (**B**) in the different treatments. Nuclei was stained with hematoxylin. Red arrows, growing follicles. All the experiments were independently repeated three times, and the

representative images are presented. Scale bars, 50 μm. Bars indicate the mean ± SD. *$p < 0.05$, **$p < 0.01$, and ***$p < 0.001$.

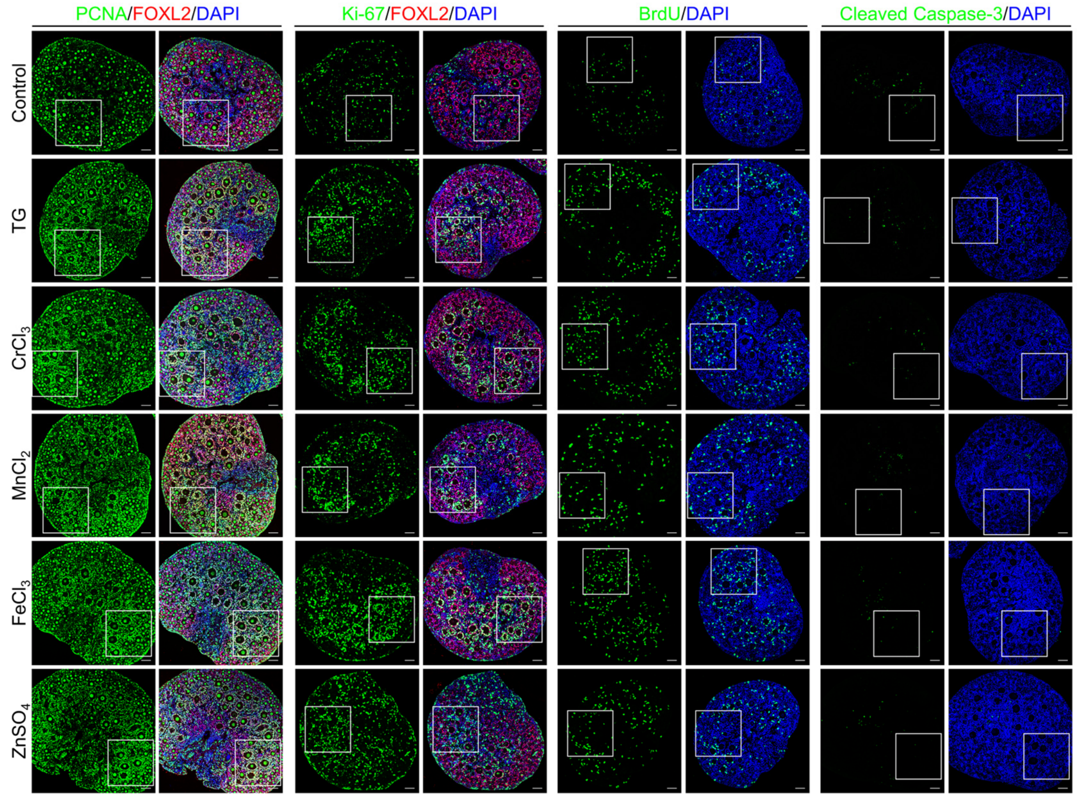

Figure S3. The metallic compounds promote the proliferation of granulosa cells in cultured mouse ovaries. The ovaries from 3 dpp mice were cultured in the medium (control), or the medium supplemented with 0.05 µM TG, 5 µM $CrCl_3$, 200 µM $MnCl_2$, 15 µM $FeCl_3$ or 35 µM $ZnSO_4$ for 2 days. Immunofluorescence staining of PCNA, Ki-67, BrdU, and Cleaved Caspase-3 (green) in the different treatments. FOXL2, red; DAPI, blue. The amplified views of the boxed area are shown in Figure 2D. Scale bars, 50 µm.

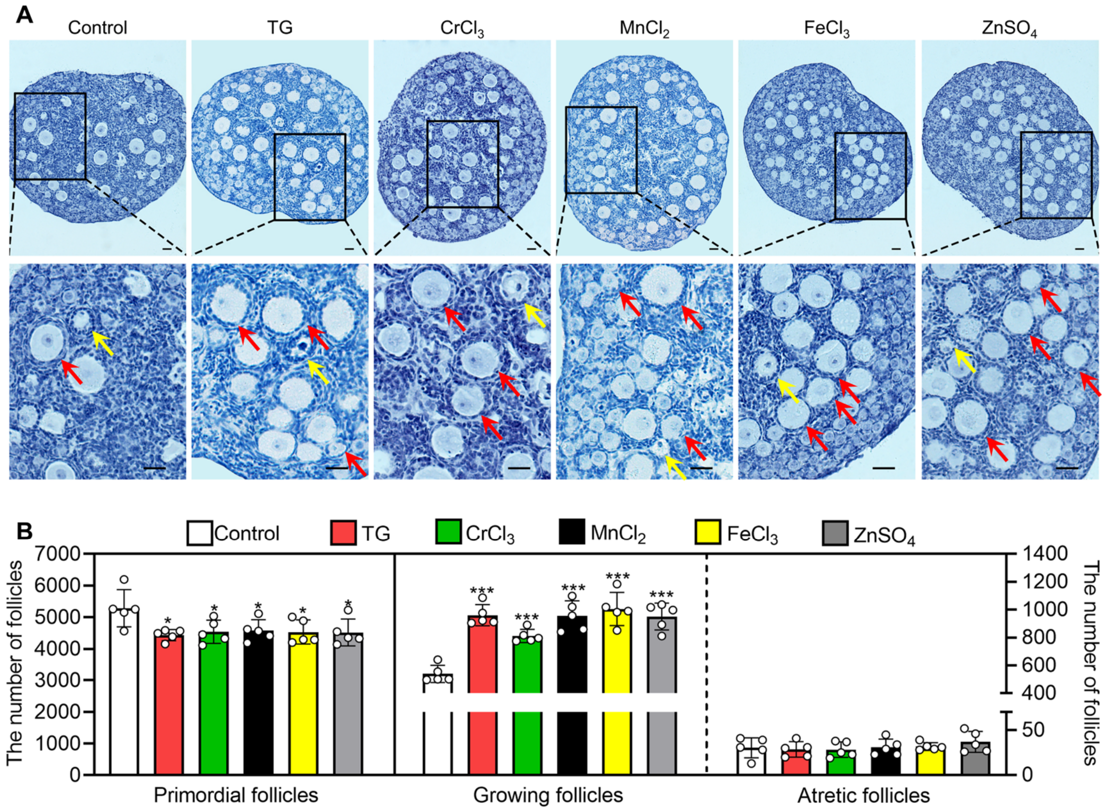

Figure S4. Subsequent development of follicles activated by the metallic compounds. The ovaries from 3 dpp mice were cultured in the medium (control), or the medium supplemented with TG, $CrCl_3$, $MnCl_2$, $FeCl_3$ or $ZnSO_4$ for 4 days and then in the drug-free medium for another 4 days. **A–B**, Morphological comparison of ovaries (**A**) and the number of primordial, growing and atretic follicles (**B**) in the different treatments. Nuclei was stained with hematoxylin. Red arrows, growing follicles; yellow arrows, atretic follicles. All the experiments were independently repeated five times, and the representative images are presented. Scale bars, 50 μm. Bars indicate the mean ± SD. *$p < 0.05$, and ***$p < 0.001$.

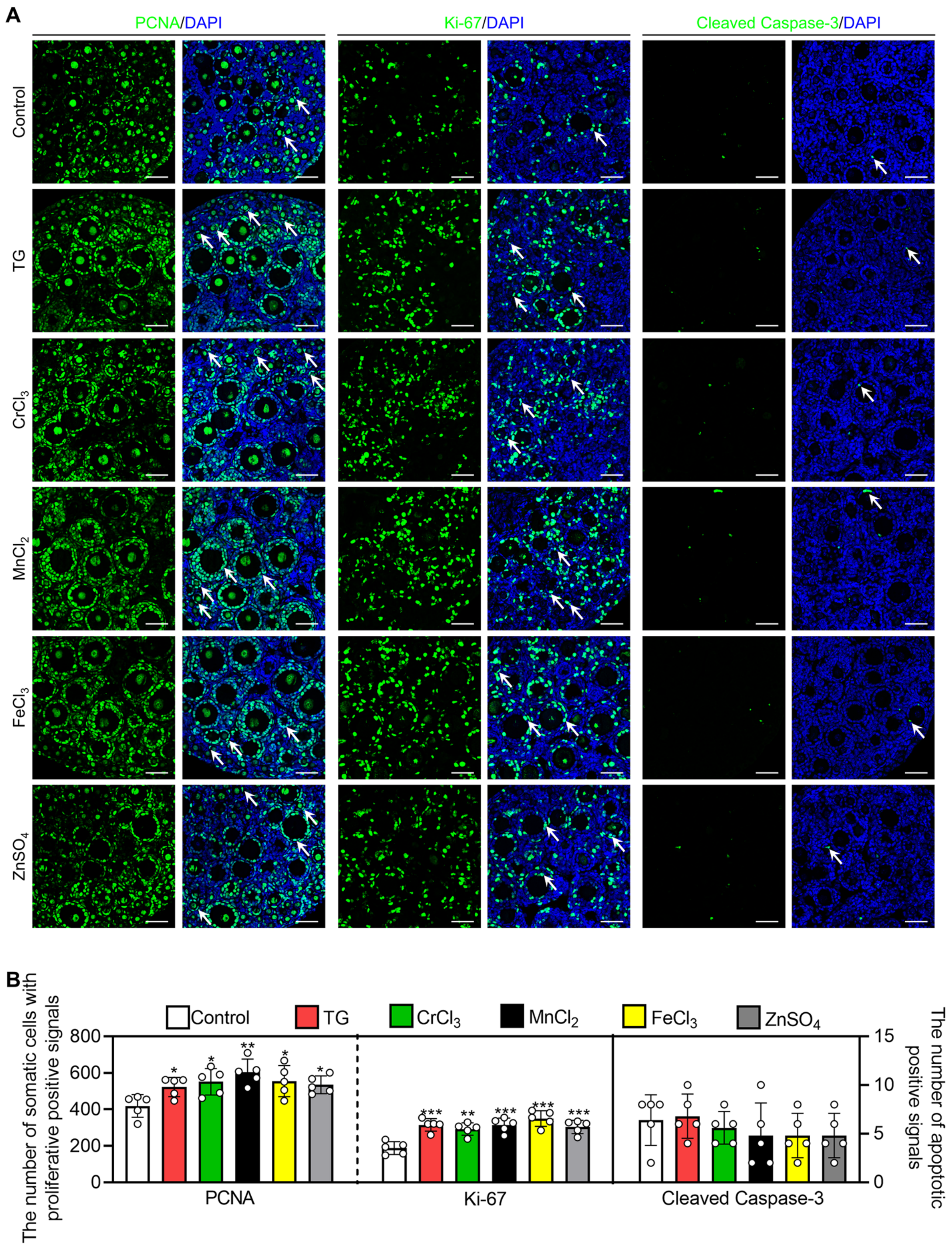

Figure S5. Effects of the metallic compounds on the development of mouse follicles. The ovaries from 3 dpp mice were cultured in the medium (control), or the medium supplemented with TG, $CrCl_3$, $MnCl_2$, $FeCl_3$ or $ZnSO_4$ for 4 days and then in the drug-free medium for another 4 days. **A**, Immunofluorescence staining of PCNA, Ki-67, and Cleaved Caspase-3 (green) in the different treatments. DAPI, blue. **B**, The

number of somatic cells with PCNA- and Ki-67-positive signals, and the number of cells with Cleaved Caspase-3-positive signals in the different treatments. All the experiments were independently repeated five times, and the representative images are presented. Scale bars, 50 μm. Bars indicate the mean ± SD. *$p < 0.05$, **$p < 0.01$, and ***$p < 0.001$.

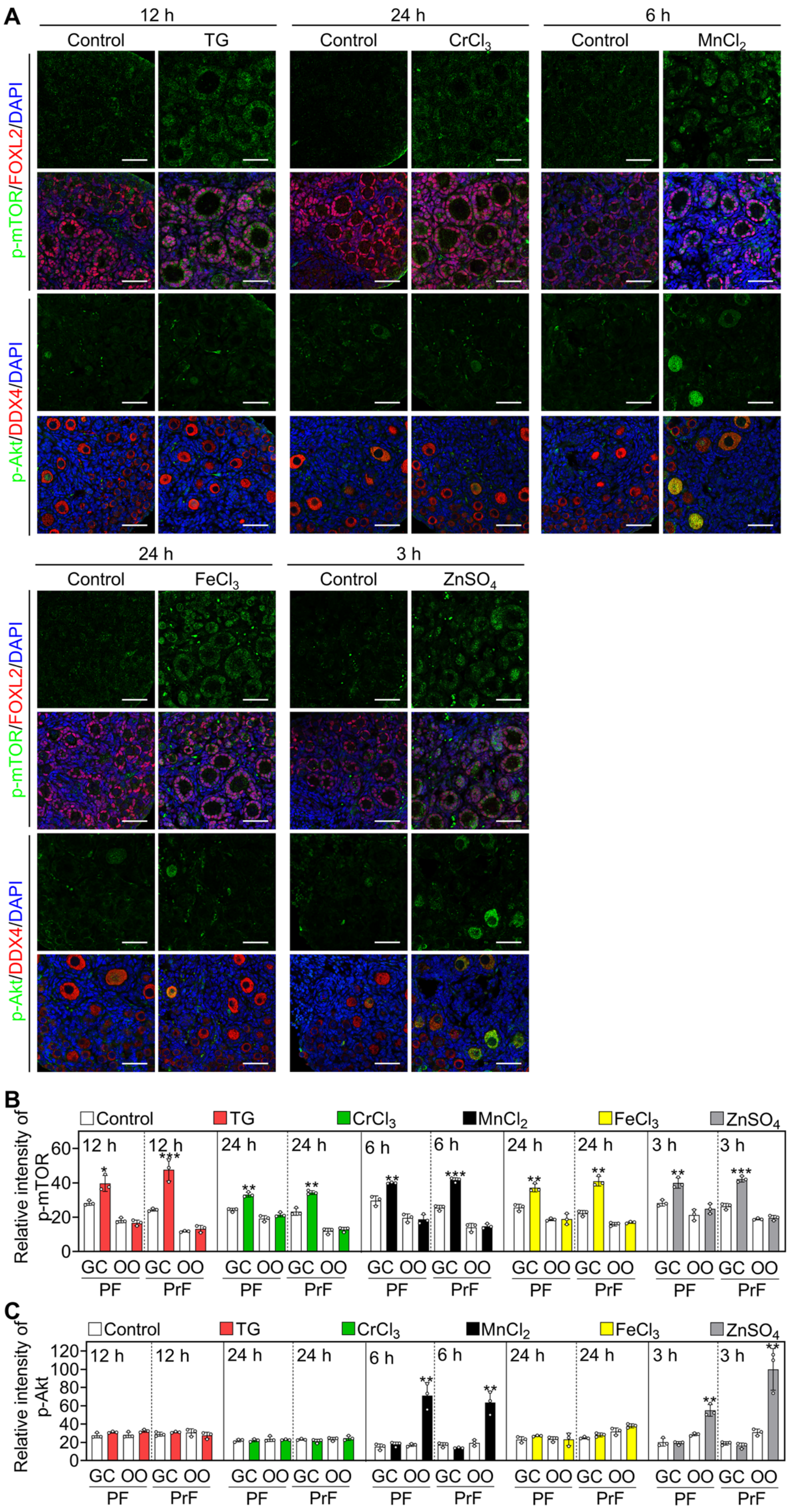

Figure S6. Effects of the metallic compounds on the intensities of p-mTOR and p-Akt fluorescent signals in cultured mouse ovaries. The ovaries from 3 dpp mice were cultured in the medium (control), or the medium supplemented with TG, $CrCl_3$,

$MnCl_2$, $FeCl_3$ or $ZnSO_4$ for indicated time. **A**, Immunofluorescence staining of p-mTOR and p-Akt (green) in the different treatments. FOXL2/DDX4, red; DAPI, blue. **B–C**, The intensities of p-mTOR (**B**) and p-Akt (**C**) fluorescent signals in granulosa cells (GC) and oocytes (OO) of primordial follicles (PF) and primary follicles (PrF). All the experiments were independently repeated three times, and the representative images are presented. Scale bars, 50 μm**.** Bars indicate the mean ± SD. $*p < 0.05$, $**p < 0.01$, and $***p < 0.001$.

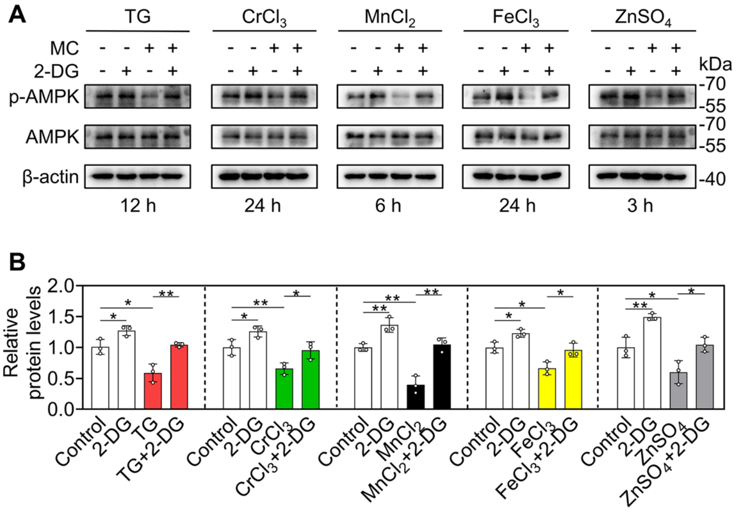

Figure S7. Effects of 2-DG on the metallic compound-decreased p-AMPK levels in cultured mouse ovaries. The ovaries from 3 dpp mice were cultured in the medium (control), or the medium supplemented with TG, $CrCl_3$, $MnCl_2$, $FeCl_3$, $ZnSO_4$ and/or 2-DG (5mM) for indicated time. **A–B**, The protein levels of p-AMPK in the different treatments. MC, metallic compounds. All the experiments were independently repeated three times, and the representative images are presented. Bars indicate the mean ± SD. $*p < 0.05$, and $**p < 0.01$.

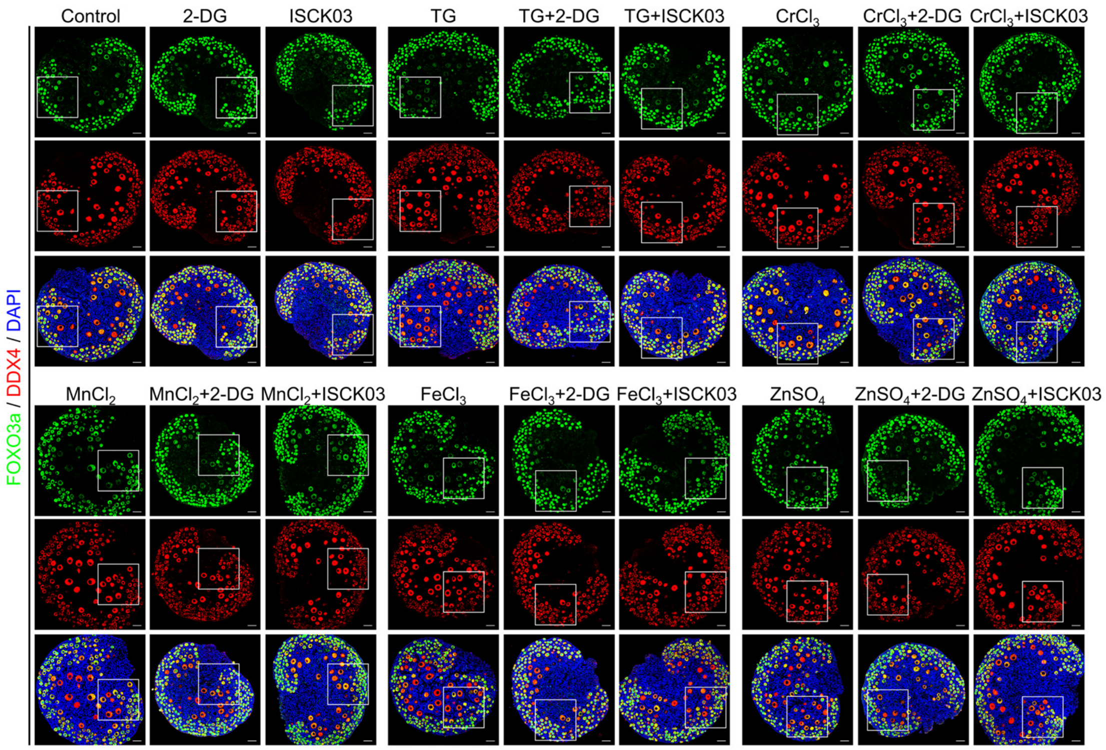

Figure S8. Effects of 2-DG and ISCK03 on the metallic compound-promoted FOXO3a nuclear export in the oocytes. The ovaries from 3 dpp mice were cultured in the medium (control), supplemented with TG, $CrCl_3$, $MnCl_2$, $FeCl_3$, $ZnSO_4$, 2-DG and/or ISCK03 (2.5 μM) for 2 days. The localization of FOXO3a (green) was shown in oocyte cytoplasm or nuclear in the different treatments. The amplified views of the boxed area are shown in Figure 4F. DDX4, red; DAPI, blue. All the experiments were independently repeated five times, and the representative images are presented. Scale bars, 50 μm.

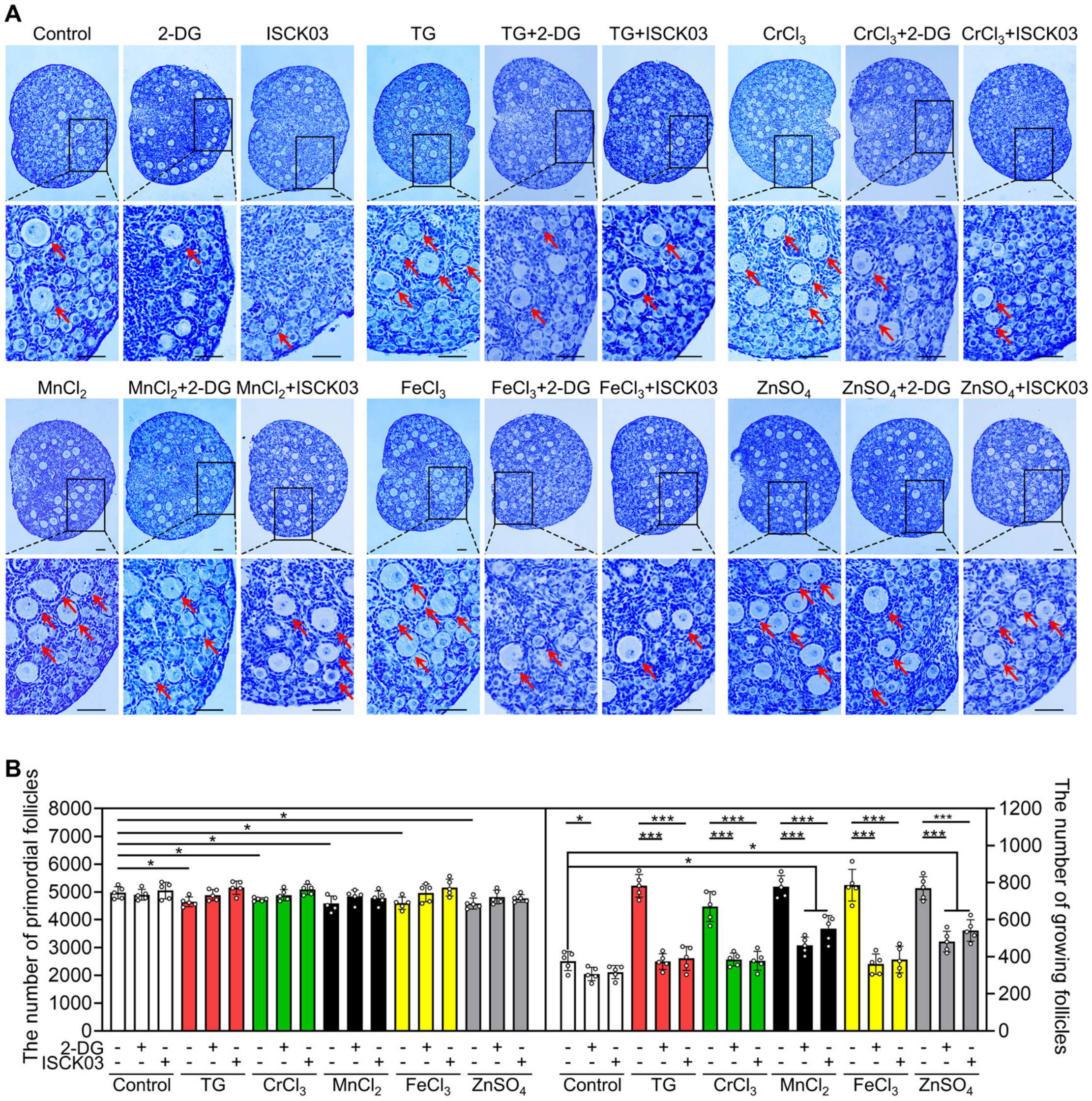

Figure S9. Effects of 2-DG and ISCK03 on the metallic compound-promoted primordial follicle activation in cultured mouse ovaries. The ovaries from 3 dpp mice were cultured in the medium (control) or the medium supplemented with TG, $CrCl_3$, $MnCl_2$, $FeCl_3$, $ZnSO_4$, 2-DG and/or ISCK03 for 2 days. **A–B**, Morphological comparison of ovaries (**A**) and the number of primordial and growing follicles (**B**) in the different treatments. Nuclei was stained with hematoxylin. Red arrows, growing follicles. All the experiments were independently repeated five times, and the representative images are presented. Scale bars, 50 µm. Bars indicate the mean ± SD. *$p < 0.05$ and ***$p < 0.001$.

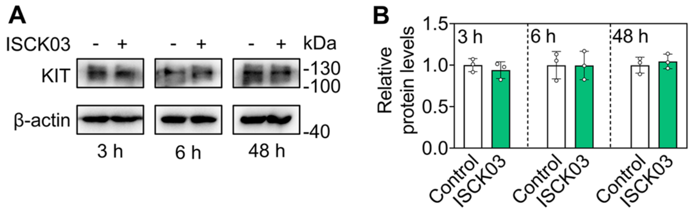

Figure S10. Effects of ISCK03 on KIT levels. The ovaries from 3 dpp mice were cultured in the medium (control), or the medium supplemented with ISCK03 for indicated time. **A–B**, The protein levels of KIT in the different time. All the experiments were independently repeated three times, and the representative images are presented. Bars indicate the mean ± SD.

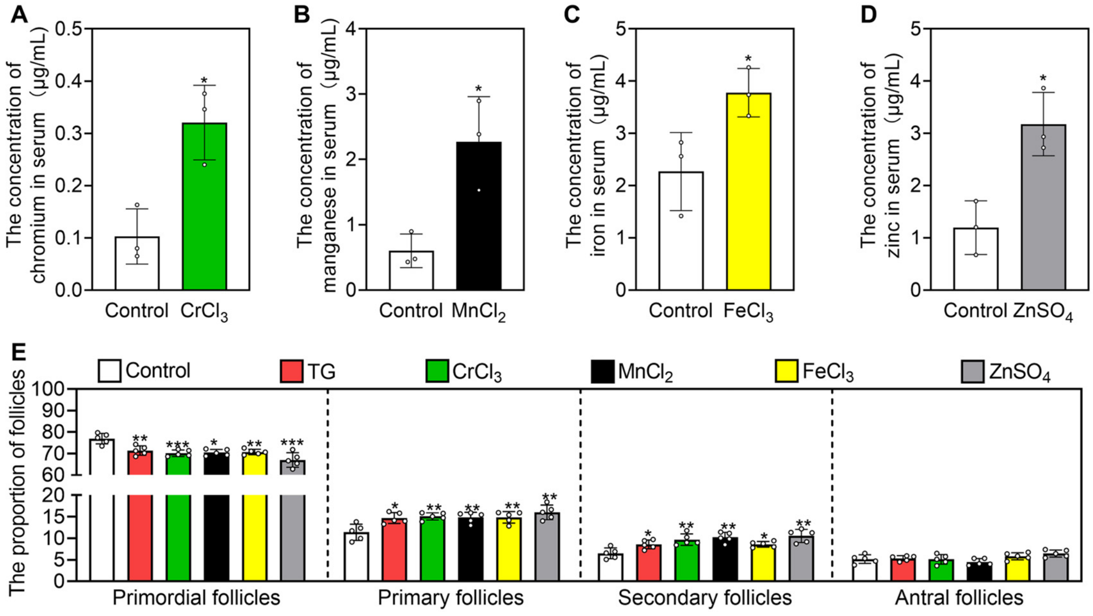

Figure S11. Effects of the metallic compounds on ion concentrations of serum and primordial follicle activation in adolescent mice. The mice were fed normal water or the water supplemented with 0.35 μM TG, 25 μM $CrCl_3$, 800 μM $MnCl_2$, 65 μM $FeCl_3$ or 200 μM $ZnSO_4$ for one week, and then the serum and ovaries were collected. **A–D**, The metal ion concentration in the serum of adolescent mice. **E**, The proportion of primordial, primary, secondary and antral follicles. All the experiments were independently repeated three or five times, and the representative images are presented. Bars indicate the mean ± SD. $*p < 0.05$, $**p < 0.01$, and $***p < 0.001$.

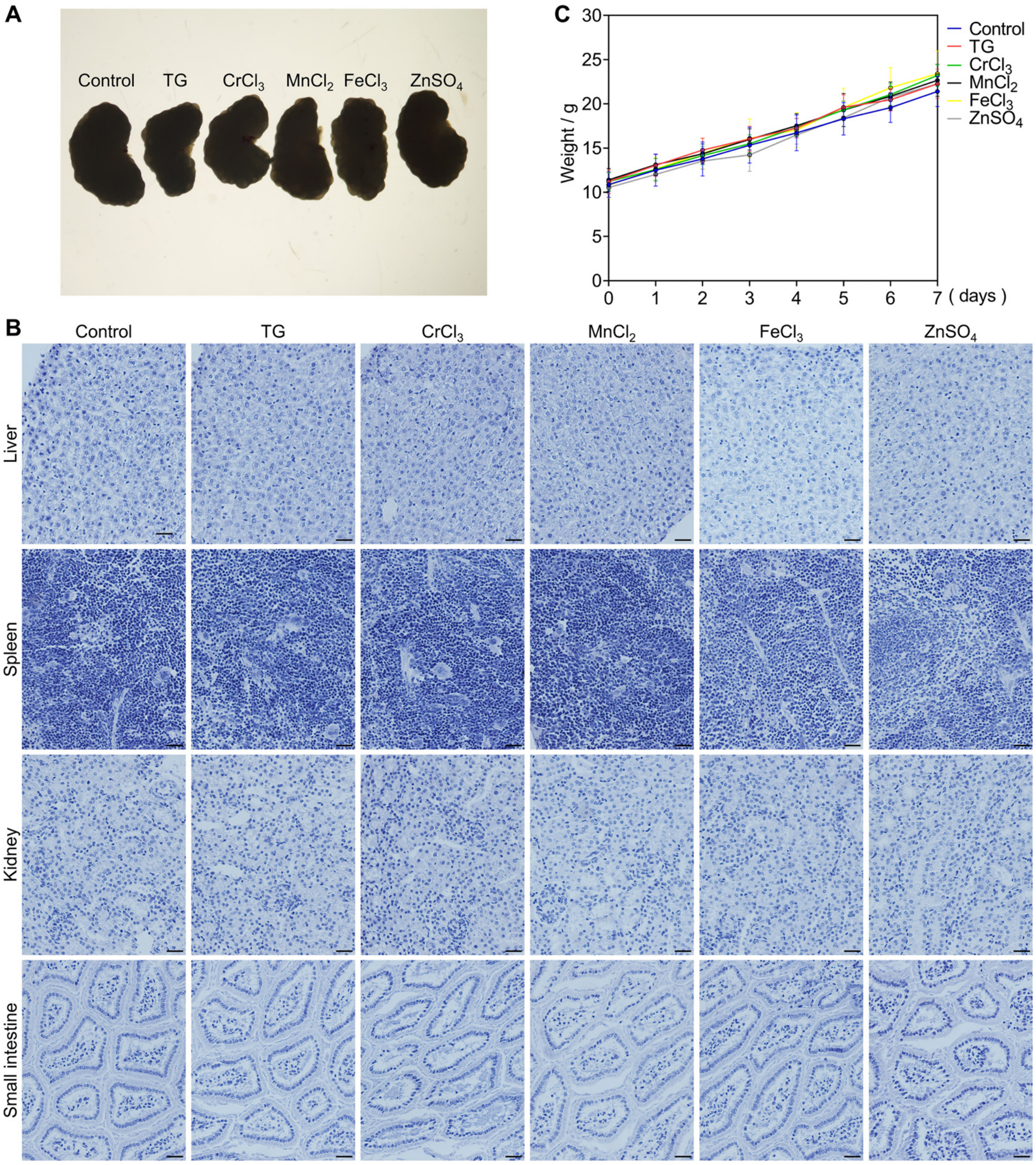

Figure S12. Effects of the metallic compounds on mouse weight and organs in adolescent mice. The mice were fed normal water or the water supplemented with TG, $CrCl_3$, $MnCl_2$, $FeCl_3$ or $ZnSO_4$ for one week, and then the organs were collected. **A**, The ovarian morphologies of adolescent mice in different treatments. **B**, Morphological comparison of liver, spleen, kidney and small intestine. **C**, The weight change in the different treatments. All the experiments were independently repeated three or five times, and the representative images are presented. Scale bars, 50 µm. Bars indicate the mean ± SD.

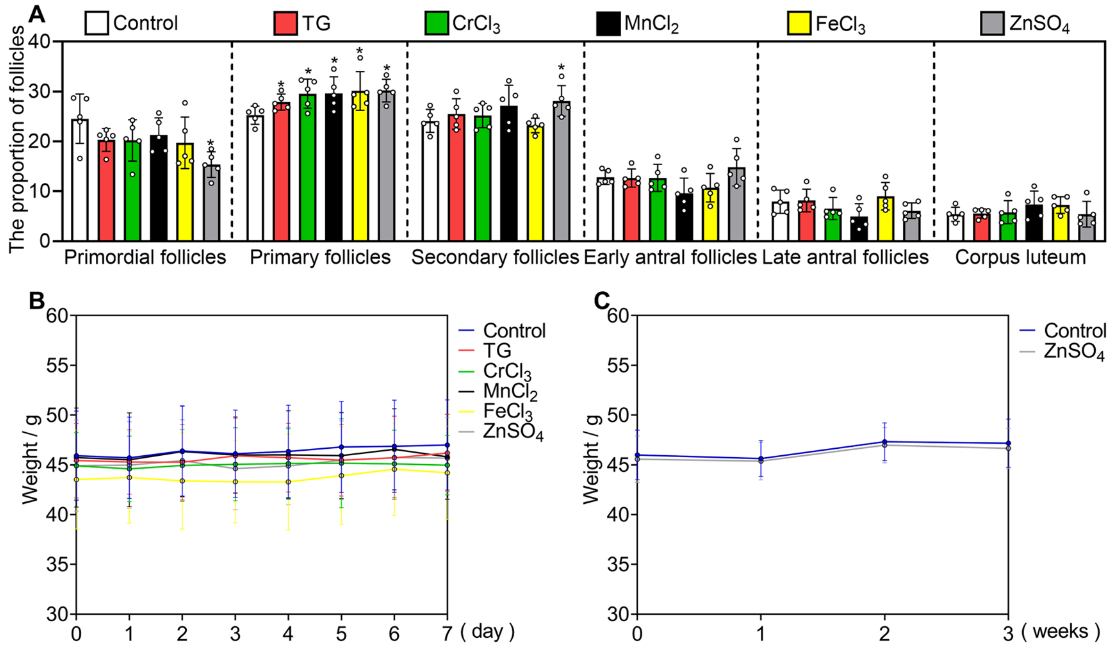

Figure S13. Effects of the metallic compounds on primordial follicle activation and mouse weight in aged mouse. The mice were fed normal water or the water supplemented with TG, $CrCl_3$, $MnCl_2$, $FeCl_3$ or $ZnSO_4$ for one week (**A–B**), and/ or then fed with normal water for further 3 weeks (**C**). **A**, The proportion of primordial, primary, secondary, early antral and late antral follicles, and corpus luteum in the different treatment of aged mice. **B–C**, The weight change in different treatments of aged mice. All the experiments were independently repeated five times. Bars indicate the mean ± SD. $*p < 0.05$.

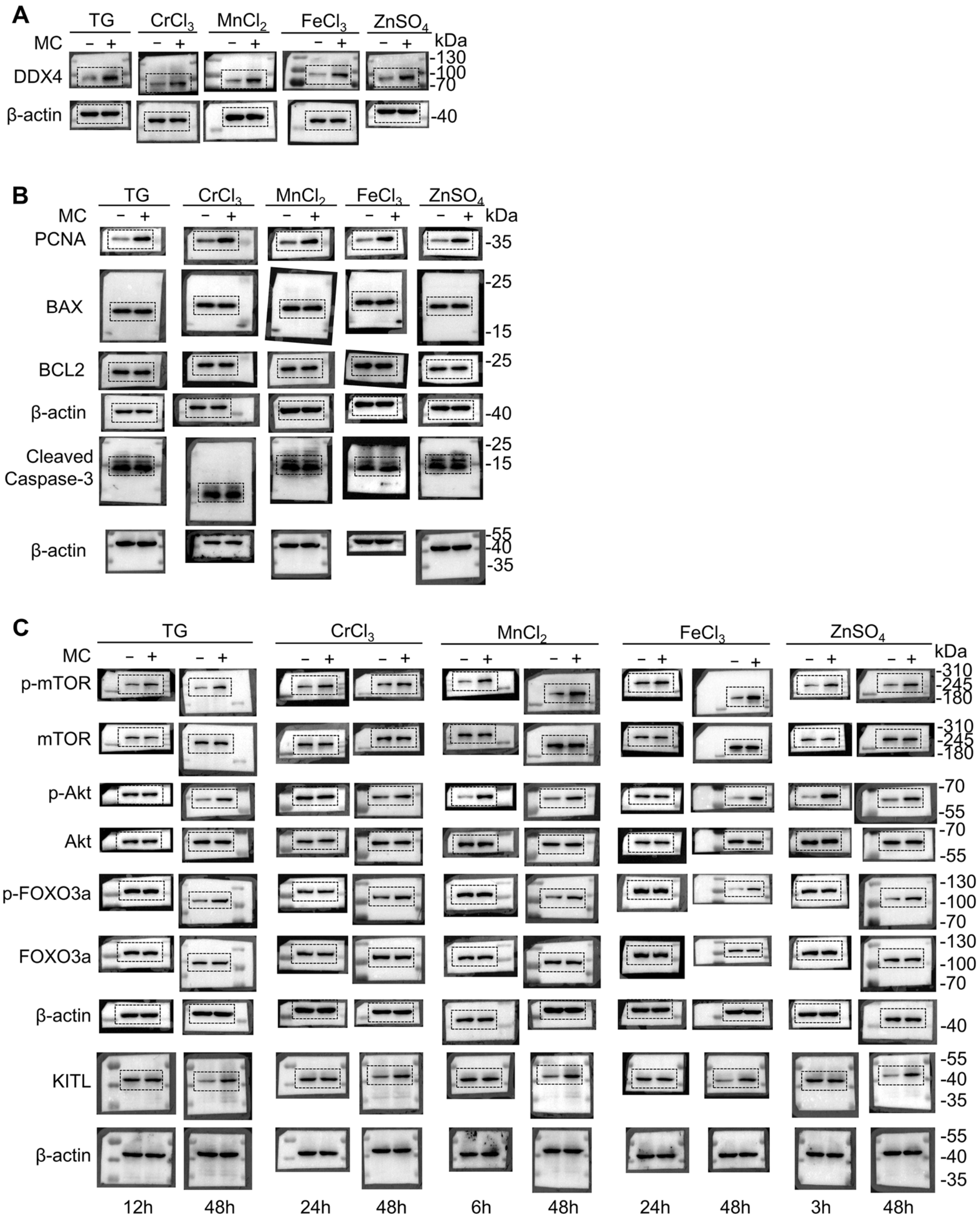

Figure S14. Uncropped scans of the western blotting results in Figure 1, 2 and 3. **A–C**, The blots in the black dashed line boxes were used in Figure 1D (**A**), 2B (**B**) and 3A (**C**).

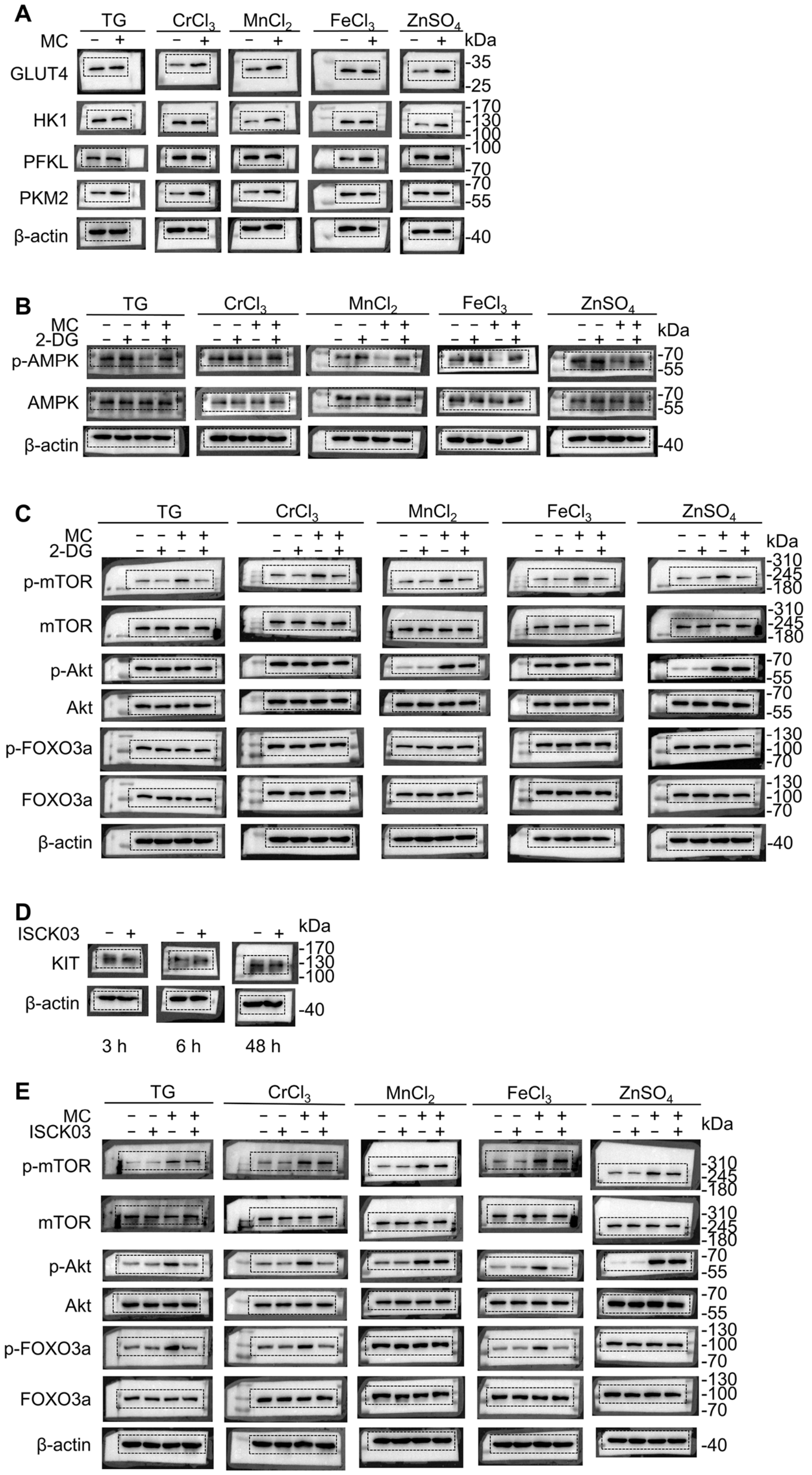

Figure S15. Uncropped scans of the western blotting results in Figure 4, S7 and S10. A–E, The blots in the black dashed line boxes were used in Figure 4A (**A**), S7A (**B**), 4B (**C**), S10A (**D**), and 4D (**E**).

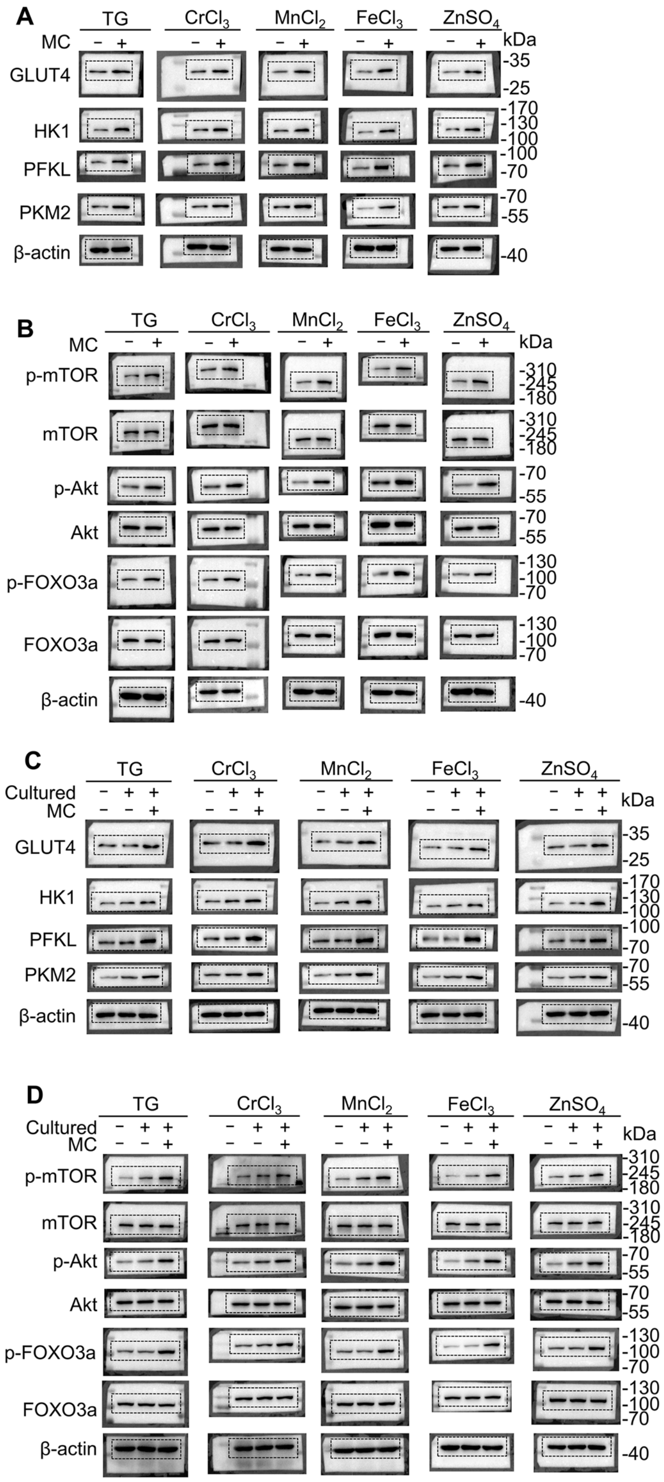

Figure S16. Uncropped scans of the western blotting results in Figure 6 and 9. A–D, The blots in the black dashed line boxes were used in Figure 6E (**A**), 6F (**B**), 9C (**C**) and 9D (**D**).

Supplementary Tables

Table S1. List of primary antibodies used in immune detection in this study.

Antibody	Catalog Code	Source	Host	Dilution	
				IF	WB
Akt	4691	Cell Signaling Technology	Rabbit	—	1:1000
p-Akt	4060	Cell Signaling Technology	Rabbit	1:200	1:1000
AMPK	ab131512	Abcam	Rabbit	—	1:1000
p-AMPK	2535	Cell Signaling Technology	Rabbit	—	1:1000
BrdU	ab1893	Abcam	Sheep	1:200	—
BAX	50599-2-Ig	Proteintech	Rabbit	—	1:1000
BCL2	26593-1-AP	Proteintech	Rabbit	—	1:1000
Cleaved Caspase-3	9664	Cell Signaling Technology	Rabbit	1:50	1:1000
DDX4	ab27591	Abcam	Mouse	1:200	1:1000
FOXL2	NB100-1277	Novus Biologicals	Goat	1:300	—
FOXO3a	12829	Cell Signaling Technology	Rabbit	1:100	1:1000
p-FOXO3a	ab26649	Abcam	Rabbit	—	1:1000
GLUT4	ab33780	Abcam	Rabbit	—	1:1000
HK1	ab150423	Abcam	Rabbit	—	1:1000
Ki-67	9129s	Cell Signaling Technology	Rabbit	1:100	—
KIT	AF1356-SP	R&D Systems	Goat	—	1:1000
KITL	sc-13126	Santa Cruz Biotechnology	Mouse	—	1:1000
mTOR	2972	Cell Signaling Technology	Rabbit	—	1:1000
p-mTOR	2971	Cell Signaling Technology	Rabbit	1:200	1:1000
PCNA	2586	Cell Signaling Technology	Mouse	1:100	1:1000
PKM2	4053	Cell Signaling Technology	Rabbit	—	1:1000
α-Tubulin	ab195887	Abcam	Mouse	1:300	—
β-actin	4967	Cell Signaling Technology	Rabbit	—	1:1000

IF: Immunofluorescence; WB: Western blotting

Table S2. Primers for qRT–PCR used in this study.

Genes	Forwards (5’-3’)	Backwards (5’-3’)
Atf5	TGGGCTGGCTCGTAGACTAT	GTCATCCAATCAGAGAAGCCG
Bax	TTTCATCCAGGATCGAGCAGG	GCAAAGTAGAAGAGGGCAACCAC
Bcl2	CTACCGTCGTGACTTCGCA	TACCCAGCCTCCGTTATCC
Caspase-3	CCGGTTACTATTCCTGGAGA	TAACACGAGTGAGGATGTGC
Eno1	TGCGTCCACTGGCATCTAC	CAGAGCAGGCGCAATAGTTTTA
Gdf9	TCTTAGTAGCCTTAGCTCTCAGG	TGTCAGTCCCATCTACAGGCA
Jak3	CCATCACGTTAGACTTTGCCA	GGCGGAGAATATAGGTGCCTG
Junb	TCACGACGACTCTTACGCAG	CCTTGAGACCCCGATAGGGA
Ki-67	ATCATTGACCGCTCCTTTAGGT	GCTCGCCTTGATGGTTCCT
Kitl	GAATCTCCGAAGAGGCCAGAA	GCTGCAACAGGGGGTAACAT
Ldha	TGTCTCCAGCAAAGACTACTGT	GACTGTACTTGACAATGTTGGGA
Mt1	AAGAGTGAGTTGGGACACCTT	CGAGACAATACAATGGCCTCC
Nucb2	GGACAAGACCAAAGTACACAACA	CCGCTCCTTATCTCCTCTATGT
P3h1	AACAGAAGTCGGAACGCGAAA	TCCACGAGGGTCTCGATCTC
Pcna	CGGCGTGAACCTGCAGAGCA	GGTTGCGGTCGCAGCGGTAT
Rpl19	CTGAAGGTCAAAGGGAATGTGTTC	TGGTCAGCCAGGAGCTTCTTG
Timp1	GCAACTCGGACCTGGTCATAA	CGGCCCGTGATGAGAAACT
Trib3	GCAAAGCGGCTGATGTCTG	AGAGTCGTGGAATGGGTATCTG
Zp3	CCTCAGGACTAACCGTGTGGA	CCATCAGGCGAAGAGAGAAAG
